# Safety profile of ipilimumab in elderly patients: a disproportionate analysis based on the FDA Adverse Event Reporting System database

**DOI:** 10.3389/fonc.2026.1689130

**Published:** 2026-06-03

**Authors:** Yang Yang, Fang Wang, Weijia Tong, Xinglan Lu, Feng Zhao, Shencun Fang, Shiqiao Wang

**Affiliations:** 1 Department of Pharmacy, Affiliated Nanjing Brain Hospital of Nanjing Medical University, Nanjing, China; 2 Department of Pharmacy, Nanjing Chest Hospital, Nanjing, China; 3 Department of Respiratory Medicine, Nanjing Chest Hospital, Nanjing, China; 4 Department of Pharmacy, The Affiliated Guangdong Second Provincial General Hospital of Jinan University, Guangzhou, China

**Keywords:** elderly patients, FAERS, immune checkpoint inhibitor, ipilimumab, pharmacovigilance

## Abstract

**Background:**

Ipilimumab is the first cytotoxic T lymphocyte protein-4 (CTLA-4) inhibitor approved in the United States. However, there is insufficient data on the safety of this drug in elderly patients. This study aimed to identify adverse events (AEs) associated with ipilimumab in elderly patients through the Adverse Event Reporting System (FAERS) database.

**Method:**

This study retrieved adverse event reports related to ipilimumab in elderly patients (≥65 years) from the first quarter of 2011 to the first quarter of 2025 in the FAERS database. Multiple disproportionality analysis methods were used to detect adverse event signals associated with ipilimumab.

**Results:**

A total of 18,698 adverse event reports were included. We identified 245 positive signals using four disproportionality methods. Common AEs included malignant neoplasm progression, diarrhea, colitis, pyrexia, rash, adrenal insufficiency, hypophysitis, and immune-mediated enterocolitis. In addition, we also identified some signals not listed on the drug label, such as orchitis, dropped head syndrome, and alopecia areata. Approximately 40% of AEs related to ipilimumab in elderly patients occurred within one month of medication use, with a median onset time of 42 days (interquartile range[IQR] 18-80days). Among these, AEs related to skin and subcutaneous tissue disorders had the shortest median time to onset, while those related to endocrine disorders had the longest median time to onset and continued to occur over time. The weight subgroup analysis indicated a statistically significant difference in the median time to AEs between the <70 kg and ≥70 kg populations (P<0.001).

**Conclusion:**

This study provides safety data on the use of ipilimumab in elderly patients and suggests prioritizing monitoring for gastrointestinal events and endocrine toxicities in elderly patients during treatment.

## 1 Introduction

Over the past decade, immune checkpoint inhibitors(ICIs) have made breakthrough progress in the treatment of various cancers, bringing new hope to many cancer patients. Currently available drugs primarily target the following therapeutic targets: programmed cell death protein 1 (PD-1), programmed death-ligand 1 (PD-L1), cytotoxic T lymphocyte associate protein-4 (CTLA-4), and lymphocyte activation gene-3 (LAG-3) (1, 2). Compared to traditional chemotherapy drugs, ICIs have shown significant survival benefits in certain cancers (3). However, it is important not to overlook the various immune-related adverse events (irAEs) caused by ICIs in clinical practice. The toxicities associated with ICIs include skin reactions, pneumonitis, thyroid dysfunction, hypophysitis, adrenal insufficiency, myocarditis, colitis, and liver toxicity (4). These AEs often lead to treatment interruption, cause tissue damage, and can be life-threatening in severe cases (5).

Ipilimumab was the first CTLA-4 inhibitor approved by the US FDA in 2011 for the treatment of unresectable or metastatic melanoma. In recent years, its approved indications have continued to expand. Besides melanoma, it is used in combination with nivolumab for the treatment of other related cancers such as renal cell carcinoma, colorectal cancer, hepatocellular carcinoma, malignant pleural mesothelioma, non-small cell lung cancer, and esophageal cancer. Currently, there is limited clinical research on ipilimumab monotherapy in elderly patients. A randomized, open-label phase III study of ipilimumab combined with nivolumab in patients aged ≥70 with advanced non-small cell lung cancer primarily reported various AEs, such as anemia, fatigue, pruritus, diarrhea, arthralgia, acute kidney injury, pneumonia, cardiotoxicity, endocrine toxicity, and colitis, among which 31% of patients experienced at least one immune-related adverse event (6). A multicenter retrospective study found that grade 3 or higher AEs of ipilimumab in elderly patients with metastatic melanoma were mainly colitis, hepatitis, and arthritis (7).However, these studies are limited by observation time and trial scale, making it difficult to detect many delayed immune-related AEs in the short term. We have noted pharmacovigilance studies on ipilimumab published in recent years; some of these studies included only a single indication, or reported differences in gender and body weight in subgroups without conducting subgroup analyses by age. In terms of data coverage, these studies only included data on the combination immunotherapy of ipilimumab and nivolumab approved by the FDA after 2015, lacking data on single immunotherapy prior to 2015 (8–11). Furthermore, off-label use may occur in clinical practice, such as the combination of ipilimumab and pembrolizumab; therefore, the aforementioned studies cannot fully assess the safety of ipilimumab in elderly patients.

Currently, the global population is rapidly aging, and cancer epidemiological statistics reveal that over half of new cancer cases and cancer-related deaths worldwide occur in patients aged ≥65 (12, 13), However, elderly patients are underrepresented in cancer trials, with statistical studies showing their participation rate at less than one-third (14). Among the FDA-approved immunotherapy registration populations during 2018-2022, patients aged ≥65 years accounted for 42.3%, with those aged ≥75 years representing only 11.1% (15).Consequently, safety data for immunotherapy in elderly patients is overall limited, and pharmacovigilance databases serve as an important supplement to safety data on ICIs use in elderly patients. The Adverse Event Reporting System(FAERS) database is one of the largest global databases for pharmacovigilance. This study collected adverse event reports of ipilimumab in elderly patients aged ≥65 years through the FAERS database and identified associated risks, aiming to provide safety references for the clinical use of ipilimumab.

## 2 Materials and methods

### 2.1 Data sources

The FAERS database is a publicly accessible free database (https://fis.fda.gov/extensions/FPD-QDE-FAERS/FPD-QDE-FAERS.html), with adverse event reports sourced from drug manufacturers, healthcare professionals, consumers, and others. FAERS data covers seven datasets: demographic and administrative information (DEMO), adverse drug reaction information (REAC), drug information (DRUG), patient outcomes information (OUTC), reported sources (RPSR), drug therapy start and end dates (THER), and indications for drug administration (INDI). A relationship was established in the FAERS database architecture that connects each data file through unique identification numbers. We downloaded data packages from the first quarter of 2011 to the first quarter of 2025 from the FAERS database and imported them into SAS 9.4 software for data cleaning. In the data, we selected the generic name and brand name of the drug, namely “ipilimumab” and “Yervoy,” for retrieval. Due to the inevitable occurrence of duplicate reports during data submission, we employed the deduplication principles recommended by the FDA to remove relevant duplicate reports: if the CASEID is the same, the most recent FDA_DT is chosen; if the CASEID and FDA_DTs are the same, the higher PRIMARYID is retained (16, 17).

In the FAERS database, drug role codes include Primary Suspect (PS), Secondary Suspect (SS), Concomitant (C), and Interacting (I) (17). The most common clinical regimen currently is ipilimumab most frequently combined with nivolumab. Considering the complexity of cancer treatment, whether early single immunotherapy or later approved dual immunotherapy, cancer treatment often involves the concurrent use of multiple other drugs. In In this study, we only included reports where ipilimumab was the PS drug and patients were aged ≥65 years. A detailed screening flowchart is provided in **Supplementary Figure 1**.

### 2.2 Data standardization and signal detection

We used the Medical Dictionary for Regulatory Activities (MedDRA) version 27.1 to code AEs for ipilimumab. This dictionary categorizes terms into five hierarchical levels from lowest to highest: Lowest Level Term (LLT), Preferred Terms (PT), High Level Term (HLT), High Level Group Term (HLGT), and System Organ Class (SOC) (18). In our study, we primarily utilized SOC and PT to classify, code, and subsequently analyze AEs associated with ipilimumab.

In pharmacovigilance studies, disproportionality analysis methods are commonly used to assess the association between drugs and AEs. In these studies, we employ various disproportionality analysis methods to detect signals related to ipilimumab, including the Reporting Odds Ratio (ROR) method, the Medicines Healthcare Products Regulatory Agency (MHRA) method, the Bayesian Confidence Propagation Neural Network (BCPNN) method, and the Multi Item Gamma Poisson Shrinker (MGPS) method. The ROR method is generally simple to calculate, with high sensitivity but low specificity. The MHRA method is an extension of the Proportional Reporting Ratio(PRR) method, offering good stability and sensitivity, though its sensitivity decreases as the number of reports increases. The BCPNN method excels at integrating multi-source data and cross-validation but involves more complex calculations. The MGPS method is particularly effective at detecting rare adverse events (19, 20). We combine these four methods to more accurately identify relevant signals while reducing bias caused by relying on a single algorithm. In this study, we define new adverse event signals as AEs not explicitly mentioned in the drug label. All algorithms are based on a 2×2 contingency table (**Supplementary Table 1**), with the relevant formulas and thresholds for each method detailed in **Supplementary Table 2**.

### 2.3 Time to onset analysis

We used the Weibull distribution test to evaluate the risk pattern of AEs with ipilimumab over time. When the shape parameter β < 1, and its 95% confidence interval is < 1, it indicates that the risk decreases over time (early failure type); when the shape parameter β equals or is close to 1, and its confidence interval includes the value 1, it indicates that the risk persists over time (random failure type); when the shape parameter β > 1, and its confidence interval does not include the value 1, it indicates that the risk increases over time (wear-out failure type) (21).

### 2.4 Statistical analysis

This study employed descriptive analysis to summarize the clinical characteristics of patients with ipilimumab-related AEs. Data management and statistical analysis were performed using Microsoft EXCEL 2024, SAS 9.4, and R 4.4.3. The Wilcoxon rank-sum test was used to compare differences in median onset time of AEs among subgroups, with P<0.05 considered statistically significant.

## 3 Results

### 3.1 Descriptive analyses

**Figure 1:**
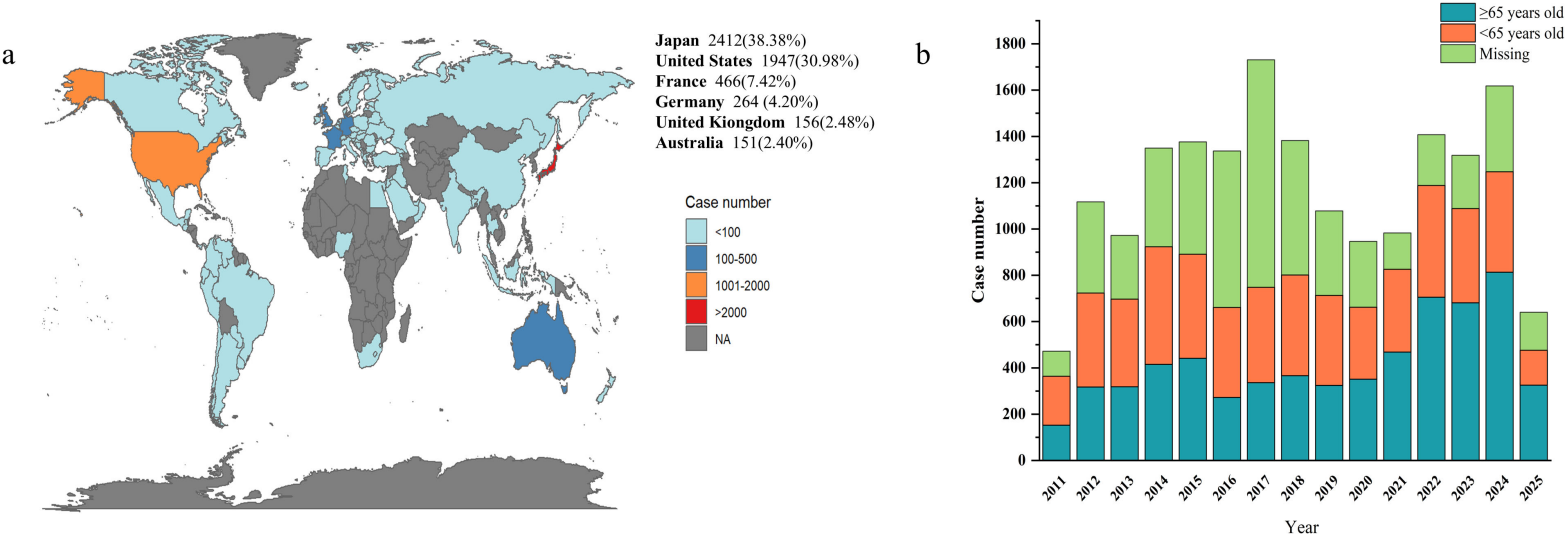
Regional and annual distribution of ipilimumab adverse events. **(a)** Regional distribution of ipilimumab adverse events. **(b)** Annual distribution of ipilimumab adverse events.

This study collected a total of 18,698 adverse event reports from the FAERS database, involving 6,284 patients. The demographic characteristics of AEs related to ipilimumab in elderly patients are shown in **Table 1**. The number of reports for male patients (69.03%) was significantly higher than that for females (29.15%). Age distribution showed that the 65–74 age group had the highest number of reported AEs (62.22%). In the weight subgroups, we found little difference in reported AEs between <70 kg and ≥70 kg. By reporting country, Japan had the highest number of reports (38.38%), followed by the United States and France. Global distribution of reporting regions showed Asia, North America, and Europe as the primary reporting areas (**Figure 1a**). Since the market launch of ipilimumab, the overall number of reports in elderly patients has shown a slow upward trend, with significantly more adverse event reports in elderly patients than in non-elderly patients in recent years (**Figure 1b**). Most reports originated from physicians, with serious adverse event reports accounting for 94.11% of the total. The main reported indications were melanoma, lung cancer, and renal cell carcinoma, comprising approximately 77% of total reports. The primary treatment outcomes for ipilimumab-related AEs in elderly patients were hospitalization (55.79%) and death (22.76%).

**Table 1:** Clinical characteristics of adverse events related to ipilimumab in the FAERS database.

Clinical characteristics	Number of events(%)
Gender	
Female	1832(29.15)
Male	4338(69.03)
Missing	114(1.81)
Age	
65-74	3910(62.22)
75-84	2116(33.67)
≥85	258(4.11)
Median(IQR)	72.00(69.00,77.00)
Weight	
<70 kg	1148(18.27)
≥70kg	1160(18.46)
Missing	3976(63.27)
Median(IQR)	70.00(58.00,83.00)
Report country or region (Top 3)	
Japan	2412(38.38)
United States	1947(30.98)
France	466(7.42)
Reporter	
Physician	2909(46.29)
Consumer	1209(19.24)
Pharmacist	995(15.83)
Other health-professional	1147(18.25)
Missing	24(0.38)
Primary outcome (Top 4)	
Hospitalization	3506(55.79)
Death	1430(22.76)
Life-Threatening	490(7.80)
Disability	129(2.05)
Indications (Top 3)	
Melanoma	2784(44.30)
Lung cancer	1225(19.49)
Renal cell carcinoma	852(13.56)
Report Type	
Serious	5914(94.11)
Non-Serious	370(5.89)

IQR, interquartile.

### 3.2 Signal detection related to SOC level

Ipilimumab-related AEs affected 26 SOC (**Supplementary Table 3**). The commonly reported AEs were gastrointestinal disorders (n=2583), general disorders and administration site conditions (n=1955), endocrine disorders (n=1344), infections and infestations (n=1251), and skin and subcutaneous tissue disorders (n=1189). Disproportionality analysis identified 4 SOC with positive signals, ranked by signal strength as: endocrine disorders (ROR: 29.04 [27.45,30.73]), hepatobiliary disorders (ROR:4.43[4.12,4.75]), neoplasms benign, malignant and unspecified (ROR:2.22 [2.09, 2.36]), and metabolism and nutrition disorders (ROR:2.21 [2.09, 2.35]).

### 3.3 Signal detection related to PT level

**Figure 2:**
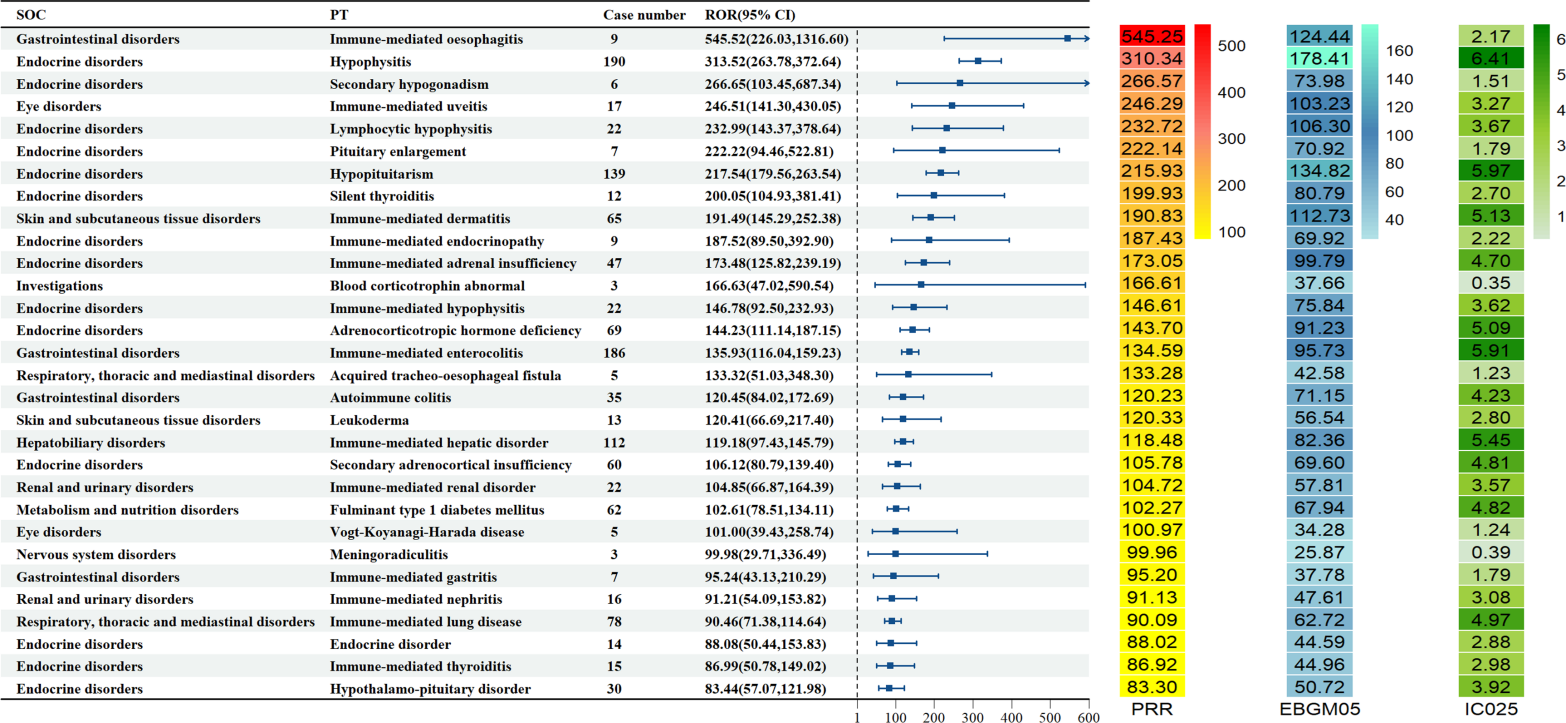
Significant signals of ipilimumab under different algorithms.

Through the FAERS database, we identified 1927 PT (**Supplementary Table 4**). Common AEs included malignant neoplasm progression, diarrhea, death, colitis, pyrexia, rash, decreased appetite, and pneumonia (**Table 2**). A total of 245 positive signals were identified using four detection methods (**Supplementary Table 5**), with common AEs including malignant neoplasm progression, diarrhea, colitis, pyrexia, rash, adrenal insufficiency, hypophysitis, and immune-mediated enterocolitis. We presented significant signals under various algorithms through forest plots and heatmaps (**Figure 2**), such as immune-mediated esophagitis, hypophysitis, hypopituitarism, secondary hypogonadism, and immune-mediated enterocolitis. Additionally, we discovered some signals not listed on drug labels, such as orchitis, dropped head syndrome, and alopecia areata.

**Table 2:** Top 50 adverse events reported for ipilimumab.

PT	Case number	ROR(95% CI)	PRR(χ^2^)	IC(IC025)	EBGM(EBGM05)
Malignant neoplasm progression*	665	16.99(15.71,18.37)	16.42(9419.00)	4.00(3.86)	16.05(14.84)
Diarrhea*	626	2.65(2.45,2.87)	2.60(620.60)	1.37(1.25)	2.59(2.39)
Death	425	1.11(1.01,1.22)	1.11(4.61)	0.15(0.01)	1.11(1.01)
Colitis*	404	33.54(30.32,37.10)	32.83(11891.80)	4.97(4.72)	31.34(28.33)
Pyrexia*	362	3.51(3.16,3.90)	3.46(634.00)	1.79(1.62)	3.45(3.11)
Rash*	329	2.73(2.44,3.04)	2.70(351.80)	1.43(1.26)	2.69(2.41)
Decreased appetite	234	2.19(1.92,2.49)	2.17(148.32)	1.12(0.92)	2.17(1.91)
Pneumonia	218	1.39(1.21,1.59)	1.38(23.36)	0.47(0.27)	1.38(1.21)
Off label use	209	0.83(0.72,0.95)	0.83(7.37)	-0.27(-0.47)	0.83(0.72)
Fatigue	206	0.88(0.77,1.01)	0.89(3.11)	-0.18(-0.38)	0.89(0.77)
Adrenal insufficiency*	200	52.31(45.27,60.45)	51.76(9240.56)	5.59(5.07)	48.10(41.63)
Hypophysitis*	190	313.52(263.78,372.64)	310.34(39973.60)	7.73(6.41)	212.06(178.41)
Immune-mediated enterocolitis*	186	135.93(116.04,159.23)	134.59(20520.50)	6.81(5.91)	112.14(95.73)
Malaise	184	1.28(1.10,1.48)	1.27(10.93)	0.35(0.13)	1.27(1.10)
Dyspnea	169	0.76(0.65,0.88)	0.76(13.20)	-0.40(-0.62)	0.76(0.65)
Nausea	166	0.75(0.64,0.88)	0.75(13.55)	-0.41(-0.63)	0.75(0.65)
Pruritus	166	1.53(1.31,1.78)	1.53(30.11)	0.61(0.38)	1.52(1.31)
Dehydration*	159	2.50(2.14,2.92)	2.48(141.05)	1.31(1.07)	2.48(2.12)
Hypothyroidism*	153	14.12(12.02,16.58)	14.01(1811.99)	3.78(3.43)	13.75(11.70)
Interstitial lung disease*	153	4.97(4.23,5.83)	4.94(477.40)	2.29(2.02)	4.91(4.18)
Hyponatremia*	152	3.92(3.34,4.60)	3.90(325.90)	1.96(1.69)	3.88(3.30)
Acute kidney injury	151	1.44(1.23,1.69)	1.44(20.08)	0.52(0.28)	1.44(1.22)
Hypopituitarism*	139	217.54(179.56,263.54)	215.93(22460.50)	7.35(5.97)	163.33(134.82)
Sepsis*	131	2.76(2.32,3.27)	2.74(144.90)	1.45(1.18)	2.74(2.30)
Vomiting	128	0.98(0.82,1.16)	0.98(0.08)	-0.04(-0.29)	0.98(0.82)
Pneumonitis*	126	10.58(8.87,12.63)	10.52(1069.30)	3.37(3.01)	10.37(8.69)
Asthenia	119	0.72(0.60,0.87)	0.72(12.55)	-0.46(-0.73)	0.72(0.61)
Cytokine release syndrome*	119	18.25(15.21,21.91)	18.14(1877.20)	4.14(3.69)	17.69(14.74)
Hepatic function abnormal*	117	7.21(6.01,8.66)	7.18(615.71)	2.83(2.49)	7.11(5.92)
Anemia	114	1.18(0.98,1.42)	1.18(3.17)	0.24(-0.03)	1.18(0.98)
Immune-mediated hepatic disorder*	112	119.18(97.43,145.79)	118.48(11077.60)	6.65(5.45)	100.74(82.36)
Liver disorder*	109	7.36(6.09,8.89)	7.32(588.74)	2.86(2.50)	7.25(6.00)
Muscular weakness	98	2.34(1.92,2.86)	2.34(74.73)	1.22(0.91)	2.33(1.91)
General physical health deterioration	91	1.82(1.48,2.24)	1.82(33.66)	0.86(0.55)	1.82(1.48)
Hypotension	91	1.01(0.82,1.24)	1.01(0.01)	0.01(-0.29)	1.01(0.82)
Pleural effusion*	89	2.87(2.33,3.54)	2.86(107.54)	1.51(1.18)	2.85(2.32)
Diabetic ketoacidosis*	86	16.98(13.70,21.04)	16.91(1255.54)	4.05(3.49)	16.51(13.32)
Hyperthyroidism*	82	17.57(14.10,21.89)	17.50(1243.22)	4.09(3.52)	17.08(13.71)
Myocarditis*	81	27.98(22.40,34.97)	27.87(2014.32)	4.74(4.02)	26.79(21.44)
Enterocolitis*	81	34.27(27.40,42.86)	34.12(2477.70)	5.02(4.23)	32.51(25.99)
Intentional product use issue*	81	3.40(2.73,4.23)	3.39(135.61)	1.75(1.39)	3.37(2.71)
Arthralgia	78	0.68(0.54,0.85)	0.68(11.92)	-0.56(-0.88)	0.68(0.54)
Immune-mediated lung disease*	78	90.46(71.38,114.64)	90.09(6053.76)	6.31(4.97)	79.48(62.72)
Alanine aminotransferase increased*	72	3.88(3.07,4.89)	3.87(152.32)	1.95(1.55)	3.85(3.05)
Febrile neutropenia*	71	2.84(2.25,3.59)	2.83(84.02)	1.50(1.12)	2.83(2.24)
Weight decreased	71	0.71(0.56,0.89)	0.71(8.62)	-0.50(-0.83)	0.71(0.56)
Aspartate aminotransferase increased*	70	3.98(3.14,5.03)	3.97(154.59)	1.98(1.58)	3.95(3.12)
Adrenocorticotropic hormone deficiency*	69	144.23(111.14,187.15)	143.70(8043.52)	6.89(5.09)	118.39(91.23)
Atrial fibrillation	68	1.05(0.83,1.33)	1.05(0.17)	0.07(-0.28)	1.05(0.83)
Platelet count decreased	67	1.41(1.11,1.79)	1.41(7.88)	0.49(0.13)	1.41(1.11)

An asterisk (*) indicates a positive signal meeting all four algorithms. ROR, reporting odds ratio; PRR, proportional reporting ratio; χ^2^, chi-squared; IC, information component; IC025, the lower limit of the 95% CI of the IC; EBGM, empirical Bayesian geometric mean; EBGM05, the lower limit of the 95% CI of EBGM.

### 3.4 Subgroup analysis

**Figure 3:**
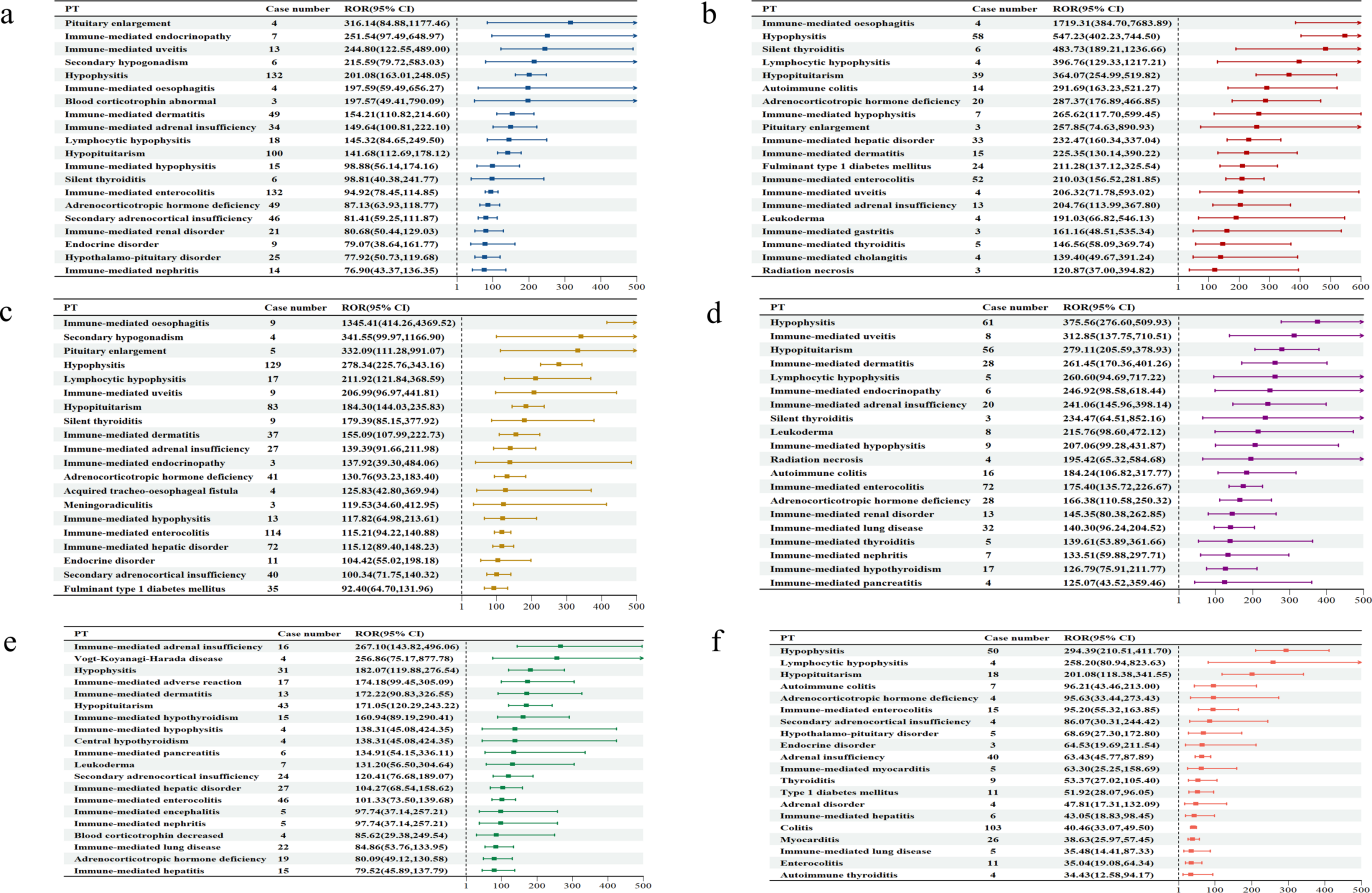
Significant signals in different subgroups.**(a)** male **(b)** female **(c)** 65–74 years old **(d)** ≥ 75 years old **(e)** <70kg **(f)** ≥70kg.

We analyzed the significant signals in the three subgroups of gender, age, and weight (**Supplementary Table 6**–**8**). **Figure 3** shows the top 20 significant signals among different subgroups. The gender subgroup showed that the top three significant signals in males were pituitary enlargement, immune-mediated endocrinopathy, and immune-mediated uveitis (**Figure 3a**), while the significant signals in female patients were immune-mediated esophagitis, hypophysitis, and silent thyroiditis (**Figure 3b**). The age subgroup showed that the significant signals in the 65–74 age group were immune-mediated esophagitis, secondary hypogonadism, and pituitary enlargement (**Figure 3c**). The significant signals in the ≥75 age group were hypophysitis, immune-mediated uveitis, and hypopituitarism (**Figure 3d**). The weight subgroup indicated that the significant signals in the <70kg group were immune-mediated adrenal insufficiency, Vogt-Koyanagi-Harada disease, and hypophysitis (**Figure 3e**), while the significant signals in the ≥70kg group were hypophysitis, lymphocytic hypophysitis, and hypopituitarism (**Figure 3f**).

### 3.5 Sensitivity analysis

Common combination medications with ipilimumab mainly include antitumor drugs such as nivolumab, carboplatin, paclitaxel, and pemetrexed. Other categories of drugs include acetaminophen, aspirin, levothyroxine, prednisone, and proton pump inhibitors. We excluded AEs related to these combination medications, and the data show that when these confounding factors are removed, AEs are significantly reduced. However, signals such as colitis, hypophysitis, myasthenia gravis, and intestinal perforation remain stable (**Supplementary Table 9**).

### 3.6 Time to onset analysis

**Figure 4:**
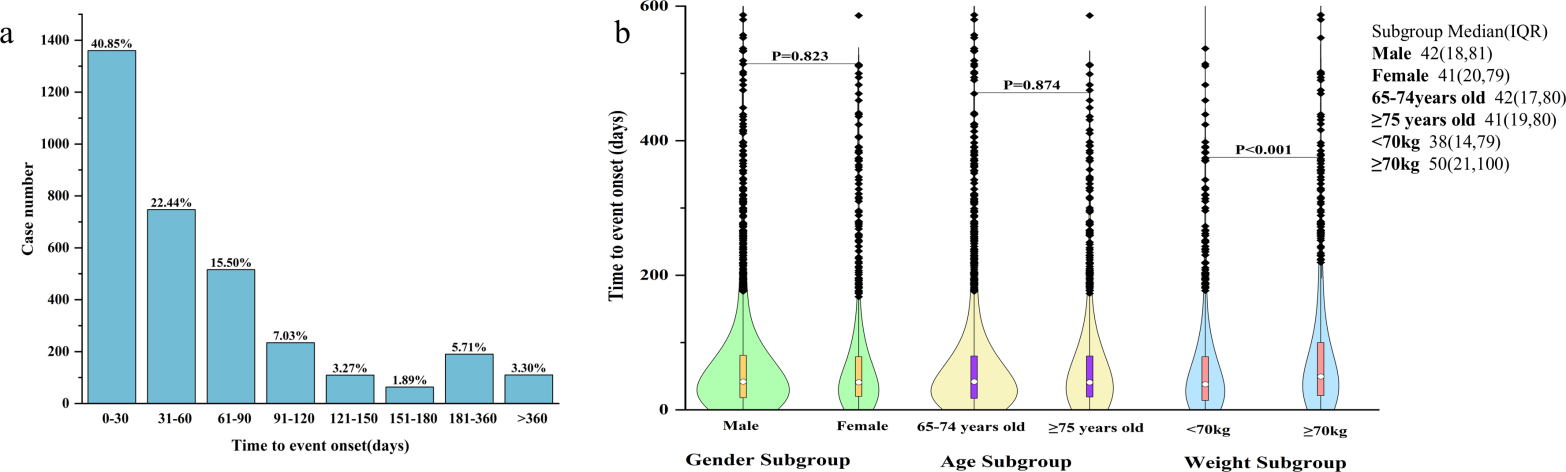
Timing of adverse events of ipilimumab. **(a)** Distribution of the timing of adverse events of ipilimumab. **(b)** Comparison of median time to onset of adverse events across different subgroups.

A total of 3,329 reports in the FAERS database documented the timing of AEs, with a median time to onset (TTO) of 42 days (IQR 18–80 days) (**Supplementary Table 10**). Approximately 40% of AEs occurred within the first month of medication use(**Figure 4a**). The Weibull distribution test indicated that the overall risk of AEs decreased over time. We analyzed the TTO at the SOC level (valid reports with adverse event occurrence time ≥ 50 cases), where endocrine disorders had the longest median TTO of 50 days, and skin and subcutaneous tissue disorders had the shortest median TTO of 19 days. The Weibull distribution test showed that the risk of endocrine disorders AEs continued over time (**Supplementary Table 10**). Additionally, we conducted separate analyses of TTO for AEs across multiple subgroups. The study showed no statistically significant difference in median TTO for AEs between different genders and ages (P > 0.05). In the weight subgroup, we found that the median TTO for AEs in the <70 kg group was earlier than that in the ≥70 kg group, with a statistically significant difference between the two median TTO (P < 0.001) (**Figure 4b**).

## 4 Discussion

We conducted a comprehensive analysis of AEs related to ipilimumab in elderly patients using the FAERS database. In our study, we found that apart from malignant neoplasm progression and death, diarrhea, colitis, pyrexia, rash, decreased appetite, pneumonia, and fatigue were common reported AEs in elderly patients, which is consistent with the drug label or certain clinical trial reports. In terms of signal strength, endocrine AEs (mainly including pituitary dysfunction and adrenal insufficiency) and gastrointestinal events (primarily various types of colitis) were particularly prominent in elderly patients. Additionally, we identified several AEs not explicitly documented in the drug label, including orchitis, dropped head syndrome, and alopecia areata, of which orchitis and alopecia areata have relevant case reports (22, 23). In this study, we also identified significant signals across different subgroups and assessed the differences in occurrence timing between subgroups. This is the largest study on AEs in the elderly population since the launch of ipilimumab. Our research provides new insights into the safety of ipilimumab use in elderly patients. Since ipilimumab may induce various AEs in elderly patients, this study focuses on some frequently reported AEs.

We noted that gastrointestinal AEs are one of the main AEs associated with ipilimumab. Studies have indicated that irAEs usually vary depending on the type of cancer, with melanoma patients being more prone to gastrointestinal AEs after ICI treatment (24). Consistent with our study, approximately 58% of gastrointestinal events occurred in melanoma patients (**Supplementary Figure 2a**). Besides common AEs in antitumor drug treatments such as diarrhea, nausea, and vomiting, various colitis induced by ipilimumab is a prominent adverse event in elderly patients. Additionally, we found some potentially fatal gastrointestinal perforation events (including intestinal perforation and large intestine perforation,n>100). A systematic review and meta-analysis showed that the incidence of ipilimumab-induced colitis ranges from 7% to 22%, significantly higher than colitis induced by PD-1/PD-L1 inhibitors. The risk of colitis is even higher when ipilimumab is combined with nivolumab (25). Wang DY et al. studied fatal toxicities related to ICIs and found that among 58 deaths caused by ipilimumab, about 40% of patients died from colitis (26). According to the statistics from this study, colitis is also one of the common AEs reported in deceased patients (**Supplementary Figure 2b**), although the data do not indicate a causal relationship between colitis and fatal outcomes. The mechanism of ipilimumab-induced colitis is not yet clear and may involve multiple pathways. Ipilimumab blocks the binding of CTLA-4 to its ligands CD80/CD86, releasing T cell inhibition, leading to excessive proliferation and activation of CD8+ and CD4+ T cells, which infiltrate the intestinal mucosa. These infiltrating activated T cells release various pro-inflammatory factors such as TNF-α, IFNγ, IL-6, and IL-17, causing intestinal inflammation and damage. Blocking CTLA-4 with ipilimumab may also impair the ability of regulatory T cells to suppress inflammatory responses. Furthermore, the microbiota is an important regulator of intestinal homeostasis and immune responses; anti-CTLA-4 therapy may lead to changes in microbial composition and reduced diversity (27–29). Clinically, the symptoms of colitis include abdominal pain, diarrhea, blood in the stool, and mucus in the stool; diarrhea is the primary symptom (27, 30). Clinicians should proactively advise elderly patients to promptly report to their physician upon experiencing any of the aforementioned symptoms or changes in bowel habits. Following relevant laboratory investigations to rule out infectious etiologies (bacterial, viral) or other causes, clinicians should further assess the severity of the condition in order to make targeted treatment decisions. In the meantime, elderly patients should maintain a lactose-free or low-fiber diet until diarrheal symptoms have resolved (31).

Endocrine dysfunction caused by ICI treatment is a common irAE. According to research findings, both in terms of the number of reports and signal strength, ipilimumab-induced endocrine AEs are particularly prominent in elderly patients. Among the endocrine AEs induced by ipilimumab, hypophysitis and hypopituitarism stand out as significant signals under various algorithms. A meta-analysis indicated that the incidence of hypophysitis after CTLA-4 inhibitor treatment is 3.2%, significantly higher than PD-1 (0.4%) and PD-L1 (<0.1%), with a 6.4% incidence in combination therapy (32). Another study reported that the rate of hypophysitis was 1.8%-3.3% with low-dose ipilimumab and 4.9%-17% with high-dose (33). The mechanism underlying ipilimumab-induced hypophysitis may involve the expression of CTLA-4 in certain cells of the pituitary gland (prolactin- and thyrotropin-secreting cells).Following administration of ipilimumab, these cells become targets for CTLA-4 antibody binding, thereby activating the complement pathway, which manifests as the deposition of C3d and C4d products. This early event resembles a Type II hypersensitivity reaction. These products damage pituitary cells and recruit macrophages and other inflammatory cells, leading to phagocytosis and enhanced antigen presentation. These subsequent events are considered characteristic of a Type IV hypersensitivity reaction, which is characterized by lymphocytic infiltration (34–36). Additionally, genetic polymorphisms in the CTLA-4 gene may increase the risk of hypophysitis (37).In this study, we also noted that adrenal insufficiency (AI) is a common endocrine event, which is a potentially life-threatening adverse event in clinical practice. According to the reports, there were fewer cases of primary adrenal insufficiency (PAI) or Addison’s disease (n=15), while secondary adrenal insufficiency (SAI) was relatively more frequent (n=60); most other reports did not distinguish between PAI and SAI. Current research indicates that ICI-induced PAI is relatively rare, whereas SAI triggered by pituitary dysfunction is more common in clinical settings, with the incidence of SAI resulting from combination therapy of ipilimumab and nivolumab reaching 6.4% (38–40). The differentiation between PAI and SAI can be achieved by monitoring morning cortisol levels, adrenocorticotropic hormone (ACTH), aldosterone, electrolytes, and other relevant indicators, further confirmed in conjunction with medical imaging (39).In addition, endocrine toxicity associated with ipilimumab includes thyroid dysfunction and diabetes. Consistent with multiple studies, our data show that thyroid dysfunction and diabetes induced by ipilimumab are not common (41, 42). Clinically noteworthy, among the top 50 AEs reported for ipilimumab, we found multiple cases of diabetic ketoacidosis, which, if not promptly managed, could seriously threaten the survival of elderly patients. Worryingly, many cancer patients exhibit symptoms such as weakness, fatigue, nausea, vomiting, sweating, and tachycardia, which are difficult to distinguish from endocrine toxicity manifestations (43). Our research indicates that endocrine AEs occur relatively late and the risk persists over time, posing a significant challenge to immunotherapy. In addition to checking blood glucose and relevant hormones at the start of treatment, clinicians should establish appropriate monitoring frequencies based on the different endocrine events and their severity following immunotherapy. Patients who develop endocrine dysfunction after receiving ICI treatment may require one or more lifelong hormone replacement therapies; therefore, elderly patients must receive adequate training to handle daily and emergency situations, including reporting early symptoms, self-monitoring, self-first aid in critical situations, and wearing medical alert bracelets (43–45).

Rash and pruritus induced by ipilimumab are the main manifestations of dermatologic AEs, and based on the timing of adverse event onset, skin AEs are also the earliest occurring irAEs. This is consistent with the conclusions of multiple studies (46, 47). Additionally, in our study, we identified the signal of alopecia areata, non-scarring hair loss associated with ipilimumab may present characteristics of alopecia areata, and CTLA-4 gene variants are linked to alopecia areata. In alopecia areata mouse models, supplementation with CTLA-4 IgG can prevent the onset of alopecia areata (48). Skin AEs from anti-CTLA-4 monotherapy or combination therapy typically affect 44%-72% of patients, with 2.4% progressing to grade 3 and 4 severe skin AEs (47). In our study, reports of severe skin AEs accounted for 10% of all skin AEs, with erythema multiforme, Stevens-Johnson syndrome, and toxic epidermal necrolysis being the main reported severe AEs (**Supplementary Figure 2c**). Except for severe skin AEs that may require temporary or permanent discontinuation of immunotherapy, most skin AEs can be managed without stopping the medication. However, many patients experience reduced willingness to continue treatment following the occurrence of skin AEs, ultimately leading to treatment discontinuation. Therefore, early dermatological assessment and interdisciplinary collaborative intervention for skin AEs can minimize their impact on patients’ quality of life, which is particularly important for maintaining continuous immunotherapy (49, 50).

In addition to the AEs mentioned above, the 50 most common AEs associated with ipilimumab, such as pneumonia, hyponatremia, interstitial lung disease, pneumonitis, acute kidney injury, immune-mediated hepatic disorder, and myocarditis, which are usually significantly associated with mortality (26, 51). Of particular concern is that early hepatotoxicity often presents with no obvious symptoms and is usually discovered incidentally during liver function monitoring. For clinicians, early identification of these toxicities has become critical for the continuation of immunotherapy. Prior to treatment, clinicians should fully inform patients of these potentially fatal risks. During the course of immunotherapy, in addition to closely observing clinical symptoms, regular hematological and imaging tests should be performed to ensure timely intervention. When the risks of treatment outweigh the benefits to the patient, permanent discontinuation of the drug or a readjustment of the treatment plan may be necessary (50). Furthermore, we found that cytokine release syndrome (CRS), which was rare in previous clinical studies, has been frequently reported. CRS is an immune overactivation phenomenon characterized by massive cytokine release, mainly manifesting as fever, fatigue, nausea, vomiting, headache, rash, and joint pain, and in severe cases can lead to organ failure and be life-threatening. CRS typically occurs with chimeric antigen receptor T-cell (CAR-T) therapy and bispecific T-cell engager (BiTE) immunotherapy. In recent years, ICI treatment has also been recognized as a cause of CRS (52, 53). A pharmacovigilance study showed that among 395 cases of CRS induced by ICIs, 35% occurred during combined treatment with nivolumab and ipilimumab (54), highlighting the need to pay special attention to CRS induced by this immune combination therapy. In this study, we found that reports of hematologic AEs in elderly patients treated with ipilimumab were not prominent, which is inconsistent with drug labels and clinical trials. This substantial difference might not genuinely reflect the actual situation; there could be various contributing factors. For instance, reporters might have selectively omitted these anticipated AEs. Also, the medication package insert or clinical trials for ipilimumab typically report AEs resulting from combination therapy, especially considering that other chemotherapy drugs might be used in cancer treatment. However, our study solely reported ipilimumab as the PS drug. Furthermore, some studies indicate a reduced risk of hematologic toxicity in elderly patients treated with ICIs (55, 56).

The global population of elderly cancer patients is gradually increasing, and the number of immunotherapy-related prescriptions is also rising sharply. Even minor AEs can lead to serious consequences in frail elderly patients. The International Society of Geriatric Oncology recommends that elderly patients undergo comprehensive assessments prior to ICI treatment, including evaluations of frailty; functional independence; fall risk and mobility; cognitive, emotional, and nutritional status; polypharmacy; social support; and comorbidities, Additionally, the patient’s willingness to undergo treatment and expected benefits should be considered (57). AEs following immunotherapy should be managed in conjunction with a multidisciplinary team approach, with emphasis on home medication education for elderly patients.

Although we conducted a comprehensive analysis of AEs related to ipilimumab in elderly patients using the FAERS database, there are certain limitations to these studies. Firstly, regarding the data source, the FAERS database is a spontaneous reporting system, which is subject to misreporting, underreporting, and selective reporting. We found a large amount of missing information on weight and dosage in the data, which prevented us from exploring the relationship between AEs and dosage or weight in depth. In terms of reporting regions, 90% of reported cases originated from Asia, North America, and Europe, which may introduce regional bias. Secondly, the data submission does not require confirmation of a causal relationship between the adverse event and the drug; the detection of positive signals only indicates a statistical association. In combination immunotherapy, it is difficult to determine whether some delayed immune-related AEs are caused by a single drug or by the combined application of drugs. Currently, the detection of new adverse event signals in pharmacovigilance often relies on human judgment, which has not yet been clearly validated. Considering the inherent limitations of FAERS, these findings should be interpreted with caution.

## 5 Conclusion

This study revealed the common AEs and potential signals of ipilimumab in elderly patients through the FAERS database. Furthermore, we have provided monitoring strategies for certain AEs; we have noted an increasing number of reports regarding life-threatening AEs, such as gastrointestinal perforation, CRS, and diabetic ketoacidosis, which require clinical vigilance. More high-quality clinical studies are needed in the future to further evaluate the safety of ipilimumab in elderly patients.

## Data Availability

The original contributions presented in the study are included in the article/**Supplementary Material**. Further inquiries can be directed to the corresponding author.
