# Supplementary material for: Safety profile of ipilimumab in elderly patients: a disproportionate analysis based on the FDA Adverse Event Reporting System database
Source: Front Oncol. 2026 Jun 3;16:1689130. doi: 10.3389/fonc.2026.1689130 (PMC13272015; doi:10.3389/fonc.2026.1689130)
Supplement: Supplementary file 1 [file DataSheet1.docx]

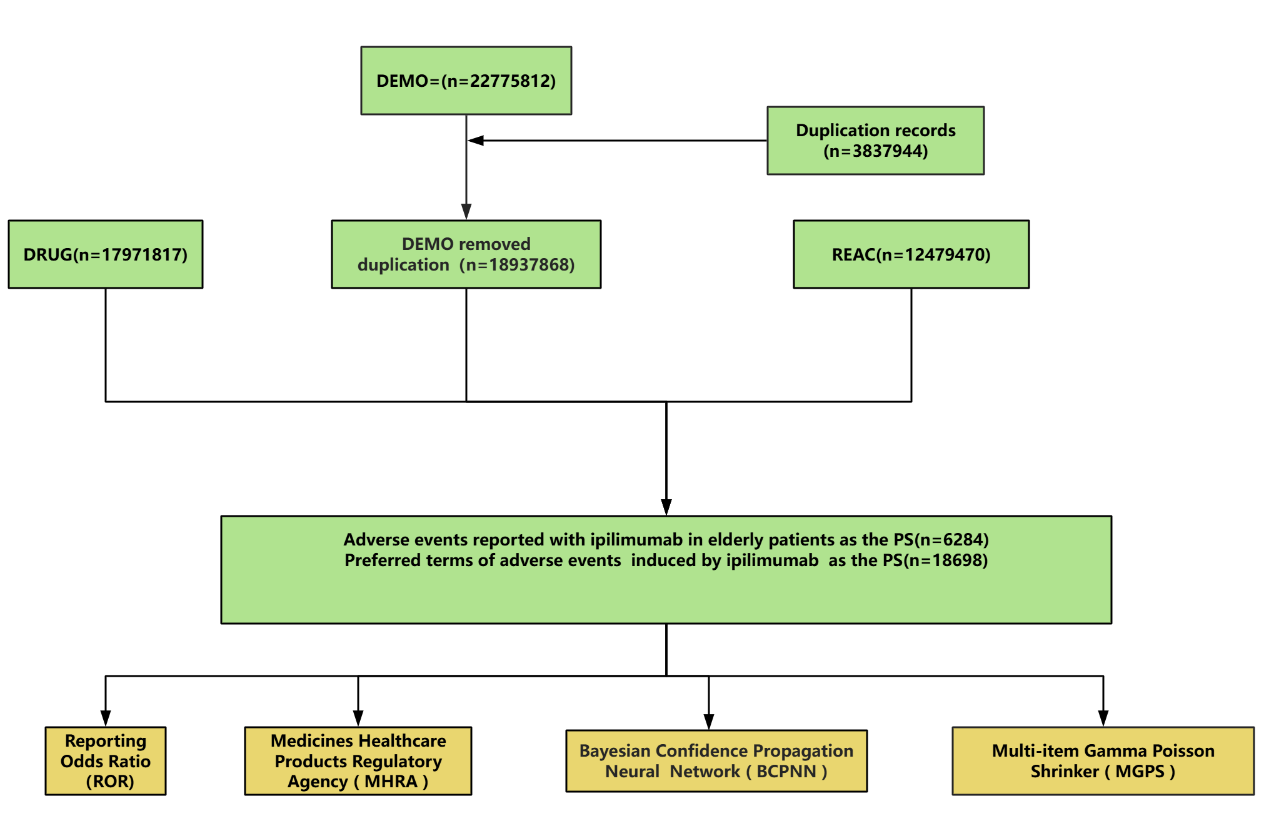


**Supplementary Figure 1 Flowchart for screening adverse events in elderly patients associated with ipilimumab**

**Supplementary Table 1 Four grid table**

|  | Target AEs | Non-target AEs | Total |
| --- | --- | --- | --- |
| Pembrolizumab | a | b | a+b |
| Non- pembrolizumab | c | d | c+d |
| Total | a+c | b+d | N=a+b+c+d |

**Supplementary Table 2 Summary of four major algorithms used for signal detection**

| Methods | Formulas | Thresholds |
| --- | --- | --- |
| ROR | $ROR=\frac{a/c}{b/d}$  $SE(lnROR)=\sqrt{(\frac{1}{a}+\frac{1}{b}+\frac{1}{c}+\frac{1}{d})}$  $95\%CI=e^{ln(ROR)\pm1.96}\sqrt{(\frac{1}{a}+\frac{1}{b}+\frac{1}{c}+\frac{1}{d})}$ | a≥3 and 95% CI＞1 |
| MHRA | $PRR=\frac{a/(a+b)}{c/(c+d)}$  $SE(lnPRR)=\sqrt{\frac{1}{a}-\frac{1}{a+b}+\frac{1}{c}-\frac{1}{c+d}}$  $95\%CI=e^{ln(PRR)\pm1.96}\sqrt{\frac{1}{a}-\frac{1}{a+b}+\frac{1}{c}-\frac{1}{c+d}}$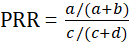  $\chi^{2}=\frac{(ad-bc)^{2}(a+b+c+d)}{(a+b)(a+c)(c+d)(b+d)}$ | a≥3 ,PRR≥2,χ²≥4 |
| BCPNN | $IC=log_{2}\frac{a(a+b+c+d)}{(a+b)(a+c)}$  ${E(IC)=log}_{2}\frac{(a+\gamma_{ij})(N+\alpha)(N+\beta)}{(N+\gamma)(a+b+\alpha_{i})(a+c+\beta_{j})}$  $V(IC)=\left( \frac{1}{\ln2} \right)^{2}[\frac{N-\alpha+\gamma-\gamma_{ij}}{(\alpha+\gamma_{ij})(1+N+\gamma)}+\frac{N-\alpha-b+\alpha-\alpha_{i}}{(\alpha+b+\alpha_{i})(1+N+\alpha)}+\frac{N-a-c+\beta-\beta_{j}}{(a+c+\beta_{i})(1+N+\beta)}]$  $\gamma=\gamma_{ij}\frac{(N+\alpha)(N+\beta)}{(a+b+a_{i})(a+c+b_{j})}$  $IC-2SD=E\left( IC \right)-2\sqrt{V\left( IC \right)}$  $\alpha_{i}=\beta_{j}=1;\alpha=\beta=2;\gamma_{ij}=1$ | IC025＞0 |
| MGPS | $EBGM=\frac{a(a+b+c+d)}{(a+c)(a+b)}$  $95\%CI=e^{ln(EBGM)\pm1.96\sqrt{(\frac{1}{a}+\frac{1}{b}+\frac{1}{c}+\frac{1}{d})}}$ | EBGM05>2 |
|  |  |  |

**Abbreviations**: N, the number of reports ; γ, γij represent the parameters of the Dirichlet distribution; α, αi, β, βj represent the parameters of the Beta distribution; SD, standard deviation; EBGM, empirical bayes geometric mean; χ2, chi-squared; IC, information component; IC025, the lower limit of 95% CI, for the IC; E(IC), the IC, expectations; V(IC), the variance of IC;EBGM05, the lower limit of the 95% CI, for EBGM.

**Table 3 Signal strength of adverse events associated with ipilimumab by System Organ Class (SOC)**

| SOC | Case number | ROR(95% CI) | PRR(χ^2^) | IC(IC025) | EBGM(EBGM05) |
| --- | --- | --- | --- | --- | --- |
| Gastrointestinal disorders | 2583 | 1.56(1.49,1.62) | 1.48(441.18) | 0.56(0.50) | 1.48(1.42) |
| General disorders and administration site conditions | 1955 | 0.62(0.59,0.65) | 0.66(405.72) | -0.60(-0.67) | 0.66(0.63) |
| Endocrine disorders﹡ | 1344 | 29.04(27.45,30.73) | 27.03(32461.70) | 4.70(4.59) | 26.01(24.58) |
| Infections and infestations | 1251 | 1.17(1.11,1.24) | 1.16(30.13) | 0.22(0.13) | 1.16(1.10) |
| Skin and subcutaneous tissue disorders | 1189 | 1.42(1.34,1.51) | 1.40(139.71) | 0.48(0.39) | 1.40(1.32) |
| Metabolism and nutrition disorders﹡ | 1170 | 2.21(2.09,2.35) | 2.14(728.39) | 1.09(1.01) | 2.14(2.01) |
| Respiratory, thoracic and mediastinal disorders | 1158 | 1.07(1.00,1.13) | 1.06(4.32) | 0.09(0.00) | 1.06(1.00) |
| Neoplasms benign, malignant and unspecified﹡ | 1134 | 2.22(2.09,2.36) | 2.15(711.75) | 1.10(1.01) | 2.14(2.02) |
| Investigations | 1077 | 0.80(0.75,0.85) | 0.81(50.33) | -0.30(-0.39) | 0.81(0.76) |
| Nervous system disorders | 963 | 0.60(0.56,0.64) | 0.62(248.32) | -0.69(-0.79) | 0.62(0.58) |
| Hepatobiliary disorders﹡ | 801 | 4.43(4.12,4.75) | 4.28(2021.08) | 2.09(1.98) | 4.26(3.97) |
| Injury, poisoning and procedural complications | 634 | 0.32(0.29,0.35) | 0.34(889.06) | -1.55(-1.66) | 0.34(0.32) |
| Musculoskeletal and connective tissue disorders | 597 | 0.60(0.55,0.65) | 0.61(152.66) | -0.70(-0.82) | 0.61(0.57) |
| Cardiac disorders | 596 | 0.88(0.81,0.96) | 0.89(8.81) | -0.17(-0.29) | 0.89(0.82) |
| Blood and lymphatic system disorders | 539 | 1.31(1.21,1.43) | 1.31(39.31) | 0.38(0.26) | 1.30(1.20) |
| Renal and urinary disorders | 535 | 1.16(1.06,1.26) | 1.15(11.39) | 0.21(0.08) | 1.15(1.06) |
| Vascular disorders | 331 | 0.67(0.60,0.75) | 0.68(52.43) | -0.56(-0.72) | 0.68(0.61) |
| Immune system disorders | 256 | 1.55(1.37,1.76) | 1.55(49.87) | 0.63(0.44) | 1.55(1.37) |
| Eye disorders | 236 | 0.60(0.53,0.69) | 0.61(60.71) | -0.72(-0.90) | 0.61(0.54) |
| Psychiatric disorders | 199 | 0.29(0.25,0.33) | 0.30(340.86) | -1.74(-1.94) | 0.30(0.26) |
| Surgical and medical procedures | 79 | 0.30(0.24,0.38) | 0.31(124.93) | -1.70(-2.01) | 0.31(0.25) |
| Ear and labyrinth disorders | 35 | 0.40(0.29,0.56) | 0.40(31.52) | -1.32(-1.78) | 0.40(0.29) |
| Reproductive system and breast disorders | 17 | 0.28(0.17,0.44) | 0.28(32.22) | -1.85(-2.47) | 0.28(0.17) |

**Table 3(Continued) Signal strength of adverse events associated with ipilimumab by System Organ Class (SOC)**

| SOC | Case number | ROR(95% CI) | PRR(χ^2^) | IC(IC025) | EBGM(EBGM05) |
| --- | --- | --- | --- | --- | --- |
| Product issues | 8 | 0.04(0.02,0.07) | 0.04(200.33) | -4.74(-5.54) | 0.04(0.02) |
| Congenital, familial and genetic disorders | 6 | 0.44(0.20,0.98) | 0.44(4.27) | -1.18(-2.15) | 0.44(0.20) |
| Social circumstances | 5 | 0.09(0.04,0.21) | 0.09(48.70) | -3.54(-4.48) | 0.09(0.04) |

Note: An asterisk (*) indicates a positive signal meeting all four algorithms. ROR, reporting odds ratio; PRR, proportional reporting ratio; χ^2^, chi-squared; IC, information component; IC025, the lower limit of the 95% CI of the IC; EBGM, empirical bayesian geometric mean; EBGM05, the lower limit of the 95% CI of EBGM.

**Supplementary Table 4 Top 100 adverse events reported for ipilimumab**

| PT | Case number | ROR(95% CI) | PRR(χ^2^) | IC(IC025) | EBGM(EBGM05) |
| --- | --- | --- | --- | --- | --- |
| Malignant neoplasm progression | 665 | 16.99(15.71,18.37) | 16.42(9419.00) | 4.00(3.86) | 16.05(14.84) |
| Diarrhoea | 626 | 2.65(2.45,2.87) | 2.60(620.60) | 1.37(1.25) | 2.59(2.39) |
| Death | 425 | 1.11(1.01,1.22) | 1.11(4.61) | 0.15(0.01) | 1.11(1.01) |
| Colitis | 404 | 33.54(30.32,37.10) | 32.83(11891.80) | 4.97(4.72) | 31.34(28.33) |
| Pyrexia | 362 | 3.51(3.16,3.90) | 3.46(634.00) | 1.79(1.62) | 3.45(3.11) |
| Rash | 329 | 2.73(2.44,3.04) | 2.70(351.80) | 1.43(1.26) | 2.69(2.41) |
| Decreased appetite | 234 | 2.19(1.92,2.49) | 2.17(148.32) | 1.12(0.92) | 2.17(1.91) |
| Pneumonia | 218 | 1.39(1.21,1.59) | 1.38(23.36) | 0.47(0.27) | 1.38(1.21) |
| Off label use | 209 | 0.83(0.72,0.95) | 0.83(7.37) | -0.27(-0.47) | 0.83(0.72) |
| Fatigue | 206 | 0.88(0.77,1.01) | 0.89(3.11) | -0.18(-0.38) | 0.89(0.77) |
| Adrenal insufficiency | 200 | 52.31(45.27,60.45) | 51.76(9240.56) | 5.59(5.07) | 48.10(41.63) |
| Hypophysitis | 190 | 313.52(263.78,372.64) | 310.34(39973.60) | 7.73(6.41) | 212.06(178.41) |
| Immune-mediated enterocolitis | 186 | 135.93(116.04,159.23) | 134.59(20520.50) | 6.81(5.91) | 112.14(95.73) |
| Malaise | 184 | 1.28(1.10,1.48) | 1.27(10.93) | 0.35(0.13) | 1.27(1.10) |
| Dyspnoea | 169 | 0.76(0.65,0.88) | 0.76(13.20) | -0.40(-0.62) | 0.76(0.65) |
| Nausea | 166 | 0.75(0.64,0.88) | 0.75(13.55) | -0.41(-0.63) | 0.75(0.65) |
| Pruritus | 166 | 1.53(1.31,1.78) | 1.53(30.11) | 0.61(0.38) | 1.52(1.31) |
| Dehydration | 159 | 2.50(2.14,2.92) | 2.48(141.05) | 1.31(1.07) | 2.48(2.12) |
| Hypothyroidism | 153 | 14.12(12.02,16.58) | 14.01(1811.99) | 3.78(3.43) | 13.75(11.70) |
| Interstitial lung disease | 153 | 4.97(4.23,5.83) | 4.94(477.40) | 2.29(2.02) | 4.91(4.18) |
| Hyponatraemia | 152 | 3.92(3.34,4.60) | 3.90(325.90) | 1.96(1.69) | 3.88(3.30) |
| Acute kidney injury | 151 | 1.44(1.23,1.69) | 1.44(20.08) | 0.52(0.28) | 1.44(1.22) |
| Hypopituitarism | 139 | 217.54(179.56,263.54) | 215.93(22460.50) | 7.35(5.97) | 163.33(134.82) |
| Sepsis | 131 | 2.76(2.32,3.27) | 2.74(144.90) | 1.45(1.18) | 2.74(2.30) |
| Vomiting | 128 | 0.98(0.82,1.16) | 0.98(0.08) | -0.04(-0.29) | 0.98(0.82) |
| Pneumonitis | 126 | 10.58(8.87,12.63) | 10.52(1069.30) | 3.37(3.01) | 10.37(8.69) |
| Asthenia | 119 | 0.72(0.60,0.87) | 0.72(12.55) | -0.46(-0.73) | 0.72(0.61) |
| Cytokine release syndrome | 119 | 18.25(15.21,21.91) | 18.14(1877.20) | 4.14(3.69) | 17.69(14.74) |
| Hepatic function abnormal | 117 | 7.21(6.01,8.66) | 7.18(615.71) | 2.83(2.49) | 7.11(5.92) |
| Anaemia | 114 | 1.18(0.98,1.42) | 1.18(3.17) | 0.24(-0.03) | 1.18(0.98) |
| Immune-mediated hepatic disorder | 112 | 119.18(97.43,145.79) | 118.48(11077.60) | 6.65(5.45) | 100.74(82.36) |
| Liver disorder | 109 | 7.36(6.09,8.89) | 7.32(588.74) | 2.86(2.50) | 7.25(6.00) |
| Muscular weakness | 98 | 2.34(1.92,2.86) | 2.34(74.73) | 1.22(0.91) | 2.33(1.91) |
| General physical health deterioration | 91 | 1.82(1.48,2.24) | 1.82(33.66) | 0.86(0.55) | 1.82(1.48) |
| Hypotension | 91 | 1.01(0.82,1.24) | 1.01(0.01) | 0.01(-0.29) | 1.01(0.82) |
| Pleural effusion | 89 | 2.87(2.33,3.54) | 2.86(107.54) | 1.51(1.18) | 2.85(2.32) |
| Diabetic ketoacidosis | 86 | 16.98(13.70,21.04) | 16.91(1255.54) | 4.05(3.49) | 16.51(13.32) |
| Hyperthyroidism | 82 | 17.57(14.10,21.89) | 17.50(1243.22) | 4.09(3.52) | 17.08(13.71) |
| Myocarditis | 81 | 27.98(22.40,34.97) | 27.87(2014.32) | 4.74(4.02) | 26.79(21.44) |
| Enterocolitis | 81 | 34.27(27.40,42.86) | 34.12(2477.70) | 5.02(4.23) | 32.51(25.99) |
| Intentional product use issue | 81 | 3.40(2.73,4.23) | 3.39(135.61) | 1.75(1.39) | 3.37(2.71) |
| Arthralgia | 78 | 0.68(0.54,0.85) | 0.68(11.92) | -0.56(-0.88) | 0.68(0.54) |
| Immune-mediated lung disease | 78 | 90.46(71.38,114.64) | 90.09(6053.76) | 6.31(4.97) | 79.48(62.72) |
| Alanine aminotransferase increased | 72 | 3.88(3.07,4.89) | 3.87(152.32) | 1.95(1.55) | 3.85(3.05) |
| Febrile neutropenia | 71 | 2.84(2.25,3.59) | 2.83(84.02) | 1.50(1.12) | 2.83(2.24) |
| Weight decreased | 71 | 0.71(0.56,0.89) | 0.71(8.62) | -0.50(-0.83) | 0.71(0.56) |
| Aspartate aminotransferase increased | 70 | 3.98(3.14,5.03) | 3.97(154.59) | 1.98(1.58) | 3.95(3.12) |
| Adrenocorticotropic hormone deficiency | 69 | 144.23(111.14,187.15) | 143.70(8043.52) | 6.89(5.09) | 118.39(91.23) |
| Atrial fibrillation | 68 | 1.05(0.83,1.33) | 1.05(0.17) | 0.07(-0.28) | 1.05(0.83) |
| Platelet count decreased | 67 | 1.41(1.11,1.79) | 1.41(7.88) | 0.49(0.13) | 1.41(1.11) |
| Abdominal pain | 65 | 1.04(0.82,1.33) | 1.04(0.11) | 0.06(-0.30) | 1.04(0.82) |
| Hepatitis | 65 | 8.08(6.32,10.32) | 8.05(396.90) | 2.99(2.49) | 7.97(6.24) |
| Immune-mediated dermatitis | 65 | 191.49(145.29,252.38) | 190.83(9542.02) | 7.22(5.13) | 148.57(112.73) |
| Headache | 64 | 0.46(0.36,0.59) | 0.47(39.60) | -1.10(-1.45) | 0.47(0.36) |
| Fulminant type 1 diabetes mellitus | 62 | 102.61(78.51,134.11) | 102.27(5390.50) | 6.47(4.82) | 88.80(67.94) |
| Thyroiditis | 61 | 65.89(50.63,85.74) | 65.67(3536.69) | 5.90(4.56) | 59.87(46.01) |
| Fall | 61 | 0.32(0.25,0.42) | 0.33(85.85) | -1.62(-1.97) | 0.33(0.25) |
| Renal failure | 61 | 1.10(0.85,1.41) | 1.10(0.53) | 0.13(-0.24) | 1.10(0.85) |
| Respiratory failure | 61 | 1.76(1.37,2.26) | 1.76(19.91) | 0.81(0.43) | 1.76(1.37) |
| Cardiac failure | 60 | 1.29(1.00,1.66) | 1.29(3.93) | 0.37(-0.01) | 1.29(1.00) |
| Secondary adrenocortical insufficiency | 60 | 106.12(80.79,139.40) | 105.78(5374.56) | 6.51(4.81) | 91.43(69.60) |
| Intestinal perforation | 60 | 15.66(12.12,20.24) | 15.61(802.02) | 3.93(3.26) | 15.28(11.82) |
| Type 1 diabetes mellitus | 60 | 45.02(34.65,58.49) | 44.88(2411.74) | 5.40(4.27) | 42.11(32.41) |
| Constipation | 57 | 0.69(0.53,0.90) | 0.69(7.88) | -0.53(-0.90) | 0.69(0.53) |
| Urinary tract infection | 57 | 0.76(0.59,0.99) | 0.76(4.13) | -0.39(-0.76) | 0.77(0.59) |
| Pulmonary toxicity | 56 | 16.57(12.70,21.61) | 16.52(796.93) | 4.01(3.29) | 16.14(12.38) |
| Thrombocytopenia | 55 | 1.16(0.89,1.52) | 1.16(1.25) | 0.22(-0.17) | 1.16(0.89) |
| Hypokalaemia | 52 | 2.23(1.70,2.93) | 2.23(35.25) | 1.16(0.72) | 2.23(1.70) |
| Hyperglycaemia | 52 | 3.76(2.86,4.94) | 3.75(104.36) | 1.90(1.43) | 3.73(2.84) |
| Septic shock | 51 | 2.63(2.00,3.46) | 2.62(51.15) | 1.39(0.94) | 2.62(1.99) |
| Prescribed overdose | 51 | 11.68(8.86,15.42) | 11.66(488.38) | 3.52(2.85) | 11.47(8.69) |
| Renal impairment | 50 | 1.25(0.95,1.65) | 1.25(2.46) | 0.32(-0.09) | 1.25(0.94) |
| Immune-mediated myocarditis | 49 | 59.85(44.67,80.20) | 59.70(2595.63) | 5.78(4.30) | 54.87(40.95) |
| Immune-mediated adrenal insufficiency | 47 | 173.48(125.82,239.19) | 173.05(6382.41) | 7.10(4.70) | 137.58(99.79) |
| Large intestine perforation | 47 | 15.02(11.25,20.06) | 14.99(600.11) | 3.88(3.09) | 14.68(10.99) |
| Back pain | 47 | 0.59(0.44,0.78) | 0.59(13.41) | -0.76(-1.16) | 0.59(0.44) |
| Skin disorder | 47 | 4.96(3.72,6.62) | 4.95(147.29) | 2.30(1.77) | 4.92(3.69) |
| C-reactive protein increased | 46 | 3.40(2.55,4.55) | 3.40(77.47) | 1.76(1.27) | 3.38(2.53) |
| Myositis | 46 | 12.89(9.63,17.27) | 12.86(493.89) | 3.66(2.91) | 12.64(9.44) |
| Hypertension | 46 | 0.61(0.45,0.81) | 0.61(11.78) | -0.72(-1.13) | 0.61(0.45) |
| Neutropenia | 45 | 0.94(0.70,1.26) | 0.94(0.19) | -0.09(-0.52) | 0.94(0.70) |
| Immune-mediated hepatitis | 45 | 57.15(42.14,77.51) | 57.01(2281.33) | 5.72(4.19) | 52.60(38.78) |
| Diabetes mellitus | 45 | 2.18(1.63,2.93) | 2.18(28.67) | 1.12(0.66) | 2.18(1.62) |
| Dizziness | 45 | 0.26(0.19,0.34) | 0.26(97.01) | -1.95(-2.36) | 0.26(0.19) |
| Neutrophil count decreased | 44 | 2.57(1.91,3.46) | 2.57(41.92) | 1.36(0.88) | 2.56(1.90) |
| Confusional state | 44 | 0.57(0.42,0.76) | 0.57(14.36) | -0.81(-1.23) | 0.57(0.42) |
| Erythema | 44 | 0.84(0.62,1.12) | 0.84(1.42) | -0.26(-0.68) | 0.84(0.62) |
| Multiple organ dysfunction syndrome | 43 | 2.30(1.70,3.10) | 2.30(31.43) | 1.20(0.72) | 2.29(1.70) |
| Neoplasm malignant | 43 | 2.23(1.65,3.01) | 2.23(29.10) | 1.15(0.68) | 2.23(1.65) |
| Rash pruritic | 43 | 2.36(1.75,3.19) | 2.36(33.54) | 1.23(0.75) | 2.35(1.74) |
| Autoimmune hepatitis | 42 | 20.31(14.94,27.62) | 20.27(746.70) | 4.30(3.33) | 19.70(14.49) |
| Tubulointerstitial nephritis | 41 | 5.36(3.94,7.29) | 5.35(143.88) | 2.41(1.82) | 5.31(3.91) |
| Pulmonary embolism | 41 | 1.29(0.95,1.75) | 1.29(2.68) | 0.37(-0.09) | 1.29(0.95) |
| Oedema peripheral | 40 | 0.70(0.51,0.96) | 0.70(5.05) | -0.51(-0.95) | 0.70(0.52) |
| Infusion related reaction | 39 | 2.71(1.98,3.72) | 2.71(41.88) | 1.43(0.92) | 2.70(1.97) |
| Drug eruption | 39 | 5.44(3.97,7.46) | 5.43(139.84) | 2.43(1.82) | 5.39(3.93) |
| Immune-mediated hypothyroidism | 38 | 81.59(58.24,114.30) | 81.43(2690.11) | 6.18(4.19) | 72.67(51.87) |
| Metastases to central nervous system | 38 | 10.12(7.34,13.94) | 10.10(306.88) | 3.32(2.55) | 9.96(7.23) |
| Condition aggravated | 37 | 0.46(0.33,0.64) | 0.46(23.08) | -1.11(-1.56) | 0.46(0.34) |
| Lipase increased | 37 | 15.43(11.13,21.38) | 15.40(487.06) | 3.91(2.98) | 15.08(10.88) |

**Supplementary Table 5 Top 100 rankings of reported positive signals for ipilimumab**

| PT | Case number | ROR(95% CI) | PRR(χ^2^) | IC(IC025) | EBGM(EBGM05) |
| --- | --- | --- | --- | --- | --- |
| Malignant neoplasm progression | 665 | 16.99(15.71,18.37) | 16.42(9419.00) | 4.00(3.86) | 16.05(14.84) |
| Diarrhoea | 626 | 2.65(2.45,2.87) | 2.60(620.60) | 1.37(1.25) | 2.59(2.39) |
| Colitis | 404 | 33.54(30.32,37.10) | 32.83(11891.8) | 4.97(4.72) | 31.34(28.33) |
| Pyrexia | 362 | 3.51(3.16,3.90) | 3.46(634.00) | 1.79(1.62) | 3.45(3.11) |
| Rash | 329 | 2.73(2.44,3.04) | 2.70(351.80) | 1.43(1.26) | 2.69(2.41) |
| Adrenal insufficiency | 200 | 52.31(45.27,60.45) | 51.76(9240.56) | 5.59(5.07) | 48.10(41.63) |
| Hypophysitis | 190 | 313.52(263.78,372.64) | 310.34(39973.60) | 7.73(6.41) | 212.06(178.41) |
| Immune-mediated enterocolitis | 186 | 135.93(116.04,159.23) | 134.59(20520.50) | 6.81(5.91) | 112.14(95.73) |
| Dehydration | 159 | 2.50(2.14,2.92) | 2.48(141.05) | 1.31(1.07) | 2.48(2.12) |
| Hypothyroidism | 153 | 14.12(12.02,16.58) | 14.01(1811.99) | 3.78(3.43) | 13.75(11.70) |
| Interstitial lung disease | 153 | 4.97(4.23,5.83) | 4.94(477.40) | 2.29(2.02) | 4.91(4.18) |
| Hyponatraemia | 152 | 3.92(3.34,4.60) | 3.90(325.90) | 1.96(1.69) | 3.88(3.30) |
| Hypopituitarism | 139 | 217.54(179.56,263.54) | 215.93(22460.50) | 7.35(5.97) | 163.33(134.82) |
| Sepsis | 131 | 2.76(2.32,3.27) | 2.74(144.90) | 1.45(1.18) | 2.74(2.30) |
| Pneumonitis | 126 | 10.58(8.87,12.63) | 10.52(1069.30) | 3.37(3.01) | 10.37(8.69) |
| Cytokine release syndrome | 119 | 18.25(15.21,21.91) | 18.14(1877.20) | 4.14(3.69) | 17.69(14.74) |
| Hepatic function abnormal | 117 | 7.21(6.01,8.66) | 7.18(615.71) | 2.83(2.49) | 7.11(5.92) |
| Immune-mediated hepatic disorder | 112 | 119.18(97.43,145.79) | 118.48(11077.60) | 6.65(5.45) | 100.74(82.36) |
| Liver disorder | 109 | 7.36(6.09,8.89) | 7.32(588.74) | 2.86(2.50) | 7.25(6.00) |
| Pleural effusion | 89 | 2.87(2.33,3.54) | 2.86(107.54) | 1.51(1.18) | 2.85(2.32) |
| Diabetic ketoacidosis | 86 | 16.98(13.70,21.04) | 16.91(1255.54) | 4.05(3.49) | 16.51(13.32) |
| Hyperthyroidism | 82 | 17.57(14.10,21.89) | 17.50(1243.22) | 4.09(3.52) | 17.08(13.71) |
| Myocarditis | 81 | 27.98(22.40,34.97) | 27.87(2014.32) | 4.74(4.02) | 26.79(21.44) |
| Enterocolitis | 81 | 34.27(27.40,42.86) | 34.12(2477.70) | 5.02(4.23) | 32.51(25.99) |
| Intentional product use issue | 81 | 3.40(2.73,4.23) | 3.39(135.61) | 1.75(1.39) | 3.37(2.71) |
| Immune-mediated lung disease | 78 | 90.46(71.38,114.64) | 90.09(6053.76) | 6.31(4.97) | 79.48(62.72) |
| Alanine aminotransferase increased | 72 | 3.88(3.07,4.89) | 3.87(152.32) | 1.95(1.55) | 3.85(3.05) |
| Febrile neutropenia | 71 | 2.84(2.25,3.59) | 2.83(84.02) | 1.50(1.12) | 2.83(2.24) |
| Aspartate aminotransferase increased | 70 | 3.98(3.14,5.03) | 3.97(154.59) | 1.98(1.58) | 3.95(3.12) |
| Adrenocorticotropic hormone deficiency | 69 | 144.23(111.14,187.15) | 143.70(8043.52) | 6.89(5.09) | 118.39(91.23) |
| Hepatitis | 65 | 8.08(6.32,10.32) | 8.05(396.90) | 2.99(2.49) | 7.97(6.24) |
| Immune-mediated dermatitis | 65 | 191.49(145.29,252.38) | 190.83(9542.02) | 7.22(5.13) | 148.57(112.73) |
| Fulminant type 1 diabetes mellitus | 62 | 102.61(78.51,134.11) | 102.27(5390.50) | 6.47(4.82) | 88.80(67.94) |
| Thyroiditis | 61 | 65.89(50.63,85.74) | 65.67(3536.69) | 5.90(4.56) | 59.87(46.01) |
| Secondary adrenocortical insufficiency | 60 | 106.12(80.79,139.40) | 105.78(5374.56) | 6.51(4.81) | 91.43(69.60) |
| Intestinal perforation | 60 | 15.66(12.12,20.24) | 15.61(802.02) | 3.93(3.26) | 15.28(11.82) |
| Type 1 diabetes mellitus | 60 | 45.02(34.65,58.49) | 44.88(2411.74) | 5.40(4.27) | 42.11(32.41) |
| Pulmonary toxicity | 56 | 16.57(12.70,21.61) | 16.52(796.93) | 4.01(3.29) | 16.14(12.38) |
| Hyperglycaemia | 52 | 3.76(2.86,4.94) | 3.75(104.36) | 1.90(1.43) | 3.73(2.84) |
| Prescribed overdose | 51 | 11.68(8.86,15.42) | 11.66(488.38) | 3.52(2.85) | 11.47(8.69) |
| Immune-mediated myocarditis | 49 | 59.85(44.67,80.20) | 59.70(2595.63) | 5.78(4.30) | 54.87(40.95) |
| Immune-mediated adrenal insufficiency | 47 | 173.48(125.82,239.19) | 173.05(6382.41) | 7.10(4.70) | 137.58(99.79) |
| Large intestine perforation | 47 | 15.02(11.25,20.06) | 14.99(600.11) | 3.88(3.09) | 14.68(10.99) |
| Skin disorder | 47 | 4.96(3.72,6.62) | 4.95(147.29) | 2.30(1.77) | 4.92(3.69) |
| C-reactive protein increased | 46 | 3.40(2.55,4.55) | 3.40(77.47) | 1.76(1.27) | 3.38(2.53) |
| Myositis | 46 | 12.89(9.63,17.27) | 12.86(493.89) | 3.66(2.91) | 12.64(9.44) |
| Immune-mediated hepatitis | 45 | 57.15(42.14,77.51) | 57.01(2281.33) | 5.72(4.19) | 52.60(38.78) |
| Autoimmune hepatitis | 42 | 20.31(14.94,27.62) | 20.27(746.70) | 4.30(3.33) | 19.70(14.49) |
| Tubulointerstitial nephritis | 41 | 5.36(3.94,7.29) | 5.35(143.88) | 2.41(1.82) | 5.31(3.91) |
| Drug eruption | 39 | 5.44(3.97,7.46) | 5.43(139.84) | 2.43(1.82) | 5.39(3.93) |
| Immune-mediated hypothyroidism | 38 | 81.59(58.24,114.30) | 81.43(2690.11) | 6.18(4.19) | 72.67(51.87) |
| Metastases to central nervous system | 38 | 10.12(7.34,13.94) | 10.10(306.88) | 3.32(2.55) | 9.96(7.23) |
| Lipase increased | 37 | 15.43(11.13,21.38) | 15.40(487.06) | 3.91(2.98) | 15.08(10.88) |
| Altered state of consciousness | 37 | 3.62(2.62,5.00) | 3.61(69.58) | 1.85(1.28) | 3.60(2.60) |
| Myasthenia gravis | 36 | 12.57(9.04,17.49) | 12.55(375.57) | 3.62(2.76) | 12.33(8.87) |
| Autoimmune colitis | 35 | 120.45(84.02,172.69) | 120.23(3505.93) | 6.67(4.23) | 102.01(71.15) |
| Erythema multiforme | 34 | 8.78(6.26,12.32) | 8.77(230.91) | 3.12(2.34) | 8.66(6.18) |
| Immune-mediated adverse reaction | 33 | 67.58(47.23,96.70) | 67.46(1962.11) | 5.94(3.95) | 61.35(42.87) |
| Uveitis | 32 | 11.46(8.08,16.26) | 11.44(299.80) | 3.49(2.60) | 11.26(7.94) |
| Drug-induced liver injury | 32 | 3.66(2.58,5.18) | 3.65(61.35) | 1.86(1.25) | 3.64(2.57) |
| Disseminated intravascular coagulation | 31 | 4.53(3.18,6.46) | 4.53(84.66) | 2.17(1.51) | 4.50(3.16) |
| Haemophagocytic lymphohistiocytosis | 31 | 13.40(9.39,19.13) | 13.38(348.16) | 3.72(2.74) | 13.14(9.20) |
| Rash maculo-papular | 31 | 3.28(2.30,4.67) | 3.28(48.84) | 1.71(1.10) | 3.27(2.29) |
| Hypothalamo-pituitary disorder | 30 | 83.44(57.07,121.98) | 83.30(2168.44) | 6.21(3.92) | 74.16(50.72) |
| Cholestasis | 30 | 3.04(2.12,4.36) | 3.04(40.89) | 1.60(0.99) | 3.03(2.12) |
| Hepatic failure | 30 | 3.00(2.10,4.30) | 3.00(39.84) | 1.58(0.97) | 2.99(2.09) |
| Encephalitis | 30 | 15.45(10.75,22.19) | 15.43(395.62) | 3.92(2.85) | 15.10(10.51) |
| Dermatitis | 28 | 5.55(3.82,8.05) | 5.54(103.35) | 2.46(1.71) | 5.50(3.79) |
| Peritonitis | 27 | 3.50(2.40,5.11) | 3.49(47.85) | 1.80(1.13) | 3.48(2.38) |
| Hypercalcaemia | 26 | 4.53(3.08,6.66) | 4.52(70.85) | 2.17(1.44) | 4.50(3.06) |
| Stevens-Johnson syndrome | 26 | 4.05(2.75,5.96) | 4.05(59.27) | 2.01(1.30) | 4.03(2.74) |
| Thyroid disorder | 25 | 5.40(3.64,8.01) | 5.40(88.82) | 2.42(1.63) | 5.36(3.62) |
| Immune-mediated myositis | 25 | 12.86(8.66,19.11) | 12.85(267.95) | 3.66(2.55) | 12.62(8.49) |
| Pemphigoid | 25 | 4.45(3.00,6.59) | 4.44(66.25) | 2.14(1.40) | 4.42(2.98) |
| Adrenal disorder | 23 | 46.50(30.47,70.98) | 46.45(956.18) | 5.44(3.36) | 43.49(28.49) |
| Hepatotoxicity | 23 | 4.39(2.91,6.61) | 4.38(59.68) | 2.12(1.34) | 4.36(2.89) |
| Lymphocytic hypophysitis | 22 | 232.99(143.37,378.64) | 232.72(3762.17) | 7.43(3.67) | 172.74(106.30) |
| Immune-mediated hypophysitis | 22 | 146.78(92.50,232.93) | 146.61(2607.92) | 6.91(3.62) | 120.35(75.84) |
| Pneumonia bacterial | 22 | 4.86(3.19,7.39) | 4.85(66.80) | 2.27(1.44) | 4.82(3.17) |
| Immune-mediated renal disorder | 22 | 104.85(66.87,164.39) | 104.72(1953.22) | 6.50(3.57) | 90.64(57.81) |
| Colitis ulcerative | 21 | 3.54(2.30,5.44) | 3.54(38.02) | 1.82(1.04) | 3.52(2.29) |
| Pneumocystis jirovecii pneumonia | 21 | 3.79(2.47,5.83) | 3.79(42.89) | 1.92(1.13) | 3.77(2.46) |
| Amylase increased | 21 | 10.53(6.84,16.20) | 10.51(178.01) | 3.37(2.24) | 10.37(6.73) |
| Facial paralysis | 21 | 5.00(3.25,7.68) | 4.99(66.54) | 2.31(1.45) | 4.96(3.23) |
| Toxic epidermal necrolysis | 21 | 3.49(2.28,5.37) | 3.49(37.16) | 1.80(1.03) | 3.48(2.27) |
| Adrenocortical insufficiency acute | 20 | 37.48(23.88,58.82) | 37.44(671.61) | 5.15(3.10) | 35.50(22.62) |
| Metastases to lung | 20 | 4.43(2.86,6.88) | 4.43(52.77) | 2.14(1.29) | 4.41(2.84) |
| Immune thrombocytopenia | 19 | 5.59(3.56,8.78) | 5.59(70.94) | 2.47(1.53) | 5.55(3.53) |
| Radiation pneumonitis | 19 | 14.19(9.01,22.36) | 14.18(227.91) | 3.80(2.43) | 13.90(8.82) |
| Cholecystitis | 18 | 4.26(2.68,6.77) | 4.26(44.55) | 2.08(1.19) | 4.23(2.66) |
| Immune-mediated uveitis | 17 | 246.51(141.30,430.05) | 246.29(3032.32) | 7.49(3.27) | 180.10(103.23) |
| Autoimmune haemolytic anaemia | 16 | 10.35(6.32,16.96) | 10.34(132.97) | 3.35(2.02) | 10.20(6.22) |
| Diarrhoea haemorrhagic | 16 | 5.68(3.47,9.30) | 5.68(61.15) | 2.50(1.44) | 5.64(3.45) |
| Cholangitis | 16 | 5.37(3.28,8.78) | 5.37(56.40) | 2.41(1.38) | 5.33(3.26) |
| Troponin increased | 16 | 4.71(2.88,7.70) | 4.71(46.40) | 2.23(1.24) | 4.68(2.86) |
| Hypoalbuminaemia | 16 | 4.64(2.84,7.59) | 4.64(45.38) | 2.21(1.22) | 4.61(2.82) |
| Immune-mediated encephalitis | 16 | 71.15(42.48,119.15) | 71.09(999.04) | 6.01(3.03) | 64.33(38.41) |
| Immune-mediated nephritis | 16 | 91.21(54.09,153.82) | 91.13(1254.76) | 6.33(3.08) | 80.29(47.61) |
| Vitiligo | 16 | 34.09(20.63,56.36) | 34.07(488.57) | 5.02(2.79) | 32.46(19.64) |
| Immune-mediated thyroiditis | 15 | 86.99(50.78,149.02) | 86.92(1127.05) | 6.27(2.98) | 77.01(44.96) |

**Supplementary Table 6 Top 30 significant adverse event signals of ipilimumab reported by gender**

| Male | | | Female | | |
| --- | --- | --- | --- | --- | --- |
| PT | Case number | ROR(95% CI) | PT | Case number | ROR(95% CI) |
| Pituitary enlargement | 4 | 316.14(84.88,1177.46) | Immune-mediated oesophagitis | 4 | 1719.31(384.70,7683.89) |
| Immune-mediated endocrinopathy | 7 | 251.54(97.49,648.97) | Hypophysitis | 58 | 547.23(402.23,744.50) |
| Immune-mediated uveitis | 13 | 244.80(122.55,489.00) | Silent thyroiditis | 6 | 483.73(189.21,1236.66) |
| Secondary hypogonadism | 6 | 215.59(79.72,583.03) | Lymphocytic hypophysitis | 4 | 396.76(129.33,1217.21) |
| Hypophysitis | 132 | 201.08(163.01,248.05) | Hypopituitarism | 39 | 364.07(254.99,519.82) |
| Immune-mediated oesophagitis | 4 | 197.59(59.49,656.27) | Autoimmune colitis | 14 | 291.69(163.23,521.27) |
| Blood corticotrophin abnormal | 3 | 197.57(49.41,790.09) | Adrenocorticotropic hormone deficiency | 20 | 287.37(176.89,466.85) |
| Immune-mediated dermatitis | 49 | 154.21(110.82,214.60) | Immune-mediated hypophysitis | 7 | 265.62(117.70,599.45) |
| Immune-mediated adrenal insufficiency | 34 | 149.64(100.81,222.10) | Pituitary enlargement | 3 | 257.85(74.63,890.93) |
| Lymphocytic hypophysitis | 18 | 145.32(84.65,249.50) | Immune-mediated hepatic disorder | 33 | 232.47(160.34,337.04) |
| Hypopituitarism | 100 | 141.68(112.69,178.12) | Immune-mediated dermatitis | 15 | 225.35(130.14,390.22) |
| Immune-mediated hypophysitis | 15 | 98.88(56.14,174.16) | Fulminant type 1 diabetes mellitus | 24 | 211.28(137.12,325.54) |
| Silent thyroiditis | 6 | 98.81(40.38,241.77) | Immune-mediated enterocolitis | 52 | 210.03(156.52,281.85) |
| Immune-mediated enterocolitis | 132 | 94.92(78.45,114.85) | Immune-mediated uveitis | 4 | 206.32(71.78,593.02) |
| Adrenocorticotropic hormone deficiency | 49 | 87.13(63.93,118.77) | Immune-mediated adrenal insufficiency | 13 | 204.76(113.99,367.80) |
| Secondary adrenocortical insufficiency | 46 | 81.41(59.25,111.87) | Leukoderma | 4 | 191.03(66.82,546.13) |
| Immune-mediated renal disorder | 21 | 80.68(50.44,129.03) | Immune-mediated gastritis | 3 | 161.16(48.51,535.34) |
| Endocrine disorder | 9 | 79.07(38.64,161.77) | Immune-mediated thyroiditis | 5 | 146.56(58.09,369.74) |
| Hypothalamo-pituitary disorder | 25 | 77.92(50.73,119.68) | Immune-mediated cholangitis | 4 | 139.40(49.67,391.24) |
| Immune-mediated nephritis | 14 | 76.90(43.37,136.35) | Radiation necrosis | 3 | 120.87(37.00,394.82) |
| Immune-mediated hyperthyroidism | 6 | 76.50(31.91,183.39) | Secondary adrenocortical insufficiency | 14 | 119.77(69.22,207.21) |
| Immune-mediated pancreatitis | 10 | 76.03(38.63,149.63) | Immune-mediated myocarditis | 13 | 118.24(66.96,208.78) |
| Immune-mediated hepatic disorder | 77 | 75.93(59.47,96.95) | Endocrine disorder | 5 | 109.30(43.85,272.43) |
| Leukoderma | 8 | 71.87(33.83,152.69) | Immune-mediated encephalitis | 5 | 107.48(43.14,267.73) |
| Immune-mediated neuropathy | 3 | 69.73(20.43,237.98) | Immune-mediated adverse reaction | 9 | 102.79(52.11,202.77) |
| Cortisol increased | 5 | 68.14(26.37,176.06) | Immune-mediated hypothyroidism | 12 | 101.28(56.24,182.37) |
| Immune-mediated lung disease | 67 | 67.87(52.35,87.99) | Immune-mediated hepatitis | 13 | 100.54(57.14,176.89) |
| Immune-mediated hypothyroidism | 26 | 67.71(44.65,102.67) | Adrenal insufficiency | 69 | 91.19(71.34,116.57) |
| Immune-mediated gastritis | 4 | 65.86(22.85,189.85) | Tri-iodothyronine free decreased | 3 | 89.95(27.90,290.01) |
| Vogt-Koyanagi-Harada disease | 3 | 65.86(19.40,223.61) | Tumour pseudoprogression | 5 | 84.85(34.31,209.80) |

**Supplementary Table 7 Top 30 significant adverse event signals of ipilimumab reported by age**

| 65-74 years old | | | ≥75 years old | | |
| --- | --- | --- | --- | --- | --- |
| PT | Case number | ROR(95% CI) | PT | Case number | ROR(95% CI) |
| Immune-mediated oesophagitis | 9 | 1345.41(414.26,4369.52) | Hypophysitis | 61 | 375.56(276.60,509.93) |
| Secondary hypogonadism | 4 | 341.55(99.97,1166.90) | Immune-mediated uveitis | 8 | 312.85(137.75,710.51) |
| Pituitary enlargement | 5 | 332.09(111.28,991.07) | Hypopituitarism | 56 | 279.11(205.59,378.93) |
| Hypophysitis | 129 | 278.34(225.76,343.16) | Immune-mediated dermatitis | 28 | 261.45(170.36,401.26) |
| Lymphocytic hypophysitis | 17 | 211.92(121.84,368.59) | Lymphocytic hypophysitis | 5 | 260.60(94.69,717.22) |
| Immune-mediated uveitis | 9 | 206.99(96.97,441.81) | Immune-mediated endocrinopathy | 6 | 246.92(98.58,618.44) |
| Hypopituitarism | 83 | 184.30(144.03,235.83) | Immune-mediated adrenal insufficiency | 20 | 241.06(145.96,398.14) |
| Silent thyroiditis | 9 | 179.39(85.15,377.92) | Silent thyroiditis | 3 | 234.47(64.51,852.16) |
| Immune-mediated dermatitis | 37 | 155.09(107.99,222.73) | Leukoderma | 8 | 215.76(98.60,472.12) |
| Immune-mediated adrenal insufficiency | 27 | 139.39(91.66,211.98) | Immune-mediated hypophysitis | 9 | 207.06(99.28,431.87) |
| Immune-mediated endocrinopathy | 3 | 137.92(39.30,484.06) | Radiation necrosis | 4 | 195.42(65.32,584.68) |
| Adrenocorticotropic hormone deficiency | 41 | 130.76(93.23,183.40) | Autoimmune colitis | 16 | 184.24(106.82,317.77) |
| Acquired tracheo-oesophageal fistula | 4 | 125.83(42.80,369.94) | Immune-mediated enterocolitis | 72 | 175.40(135.72,226.67) |
| Meningoradiculitis | 3 | 119.53(34.60,412.95) | Adrenocorticotropic hormone deficiency | 28 | 166.38(110.58,250.32) |
| Immune-mediated hypophysitis | 13 | 117.82(64.98,213.61) | Immune-mediated renal disorder | 13 | 145.35(80.38,262.85) |
| Immune-mediated enterocolitis | 114 | 115.21(94.22,140.88) | Immune-mediated lung disease | 32 | 140.30(96.24,204.52) |
| Immune-mediated hepatic disorder | 72 | 115.12(89.40,148.23) | Immune-mediated thyroiditis | 5 | 139.61(53.89,361.66) |
| Endocrine disorder | 11 | 104.42(55.02,198.18) | Immune-mediated nephritis | 7 | 133.51(59.88,297.71) |
| Secondary adrenocortical insufficiency | 40 | 100.34(71.75,140.32) | Immune-mediated hypothyroidism | 17 | 126.79(75.91,211.77) |
| Fulminant type 1 diabetes mellitus | 35 | 92.40(64.70,131.96) | Immune-mediated pancreatitis | 4 | 125.07(43.52,359.46) |
| Autoimmune colitis | 19 | 90.24(55.69,146.24) | Immune-mediated hepatic disorder | 40 | 123.73(88.58,172.83) |
| Vogt-Koyanagi-Harada disease | 3 | 89.65(26.64,301.73) | Fulminant type 1 diabetes mellitus | 27 | 119.63(79.74,179.47) |
| Immune-mediated gastritis | 5 | 87.90(34.37,224.80) | Secondary adrenocortical insufficiency | 20 | 112.73(70.50,180.24) |
| Hypothalamo-pituitary disorder | 18 | 78.05(47.75,127.60) | Immune-mediated cholangitis | 6 | 106.62(45.42,250.28) |
| Immune-mediated renal disorder | 9 | 76.88(38.40,153.94) | Immune-mediated encephalitis | 5 | 102.87(40.48,261.43) |
| Central hypothyroidism | 5 | 76.63(30.20,194.47) | Immune-mediated hyperthyroidism | 3 | 97.70(29.41,324.52) |
| Immune-mediated nephritis | 9 | 70.81(35.48,141.34) | Hypothalamo-pituitary disorder | 12 | 92.07(50.60,167.52) |
| Leukoderma | 5 | 69.51(27.53,175.51) | Immune-mediated myocarditis | 26 | 74.95(50.09,112.15) |
| Immune-mediated lung disease | 46 | 69.33(51.06,94.12) | Immune-mediated hepatitis | 16 | 74.13(44.37,123.84) |
| Immune-mediated thyroiditis | 10 | 68.74(35.71,132.29) | Vitiligo | 9 | 74.11(37.39,146.86) |

**Supplementary Table 8 Top 30 significant adverse event signals of ipilimumab reported by weight**

| ＜70kg | | | ≥70kg | | |
| --- | --- | --- | --- | --- | --- |
| PT | Case number | ROR(95% CI) | PT | Case number | ROR(95% CI) |
| Immune-mediated adrenal insufficiency | 16 | 267.10(143.82,496.06) | Hypophysitis | 50 | 294.39(210.51,411.70) |
| Vogt-Koyanagi-Harada disease | 4 | 256.86(75.17,877.78) | Lymphocytic hypophysitis | 4 | 258.20(80.94,823.63) |
| Hypophysitis | 31 | 182.07(119.88,276.54) | Hypopituitarism | 18 | 201.08(118.38,341.55) |
| Immune-mediated adverse reaction | 17 | 174.18(99.45,305.09) | Autoimmune colitis | 7 | 96.21(43.46,213.00) |
| Immune-mediated dermatitis | 13 | 172.22(90.83,326.55) | Adrenocorticotropic hormone deficiency | 4 | 95.63(33.44,273.43) |
| Hypopituitarism | 43 | 171.05(120.29,243.22) | Immune-mediated enterocolitis | 15 | 95.20(55.32,163.85) |
| Immune-mediated hypothyroidism | 15 | 160.94(89.19,290.41) | Secondary adrenocortical insufficiency | 4 | 86.07(30.31,244.42) |
| Immune-mediated hypophysitis | 4 | 138.31(45.08,424.35) | Hypothalamo-pituitary disorder | 5 | 68.69(27.30,172.80) |
| Central hypothyroidism | 4 | 138.31(45.08,424.35) | Endocrine disorder | 3 | 64.53(19.69,211.54) |
| Immune-mediated pancreatitis | 6 | 134.91(54.15,336.11) | Adrenal insufficiency | 40 | 63.43(45.77,87.89) |
| Leukoderma | 7 | 131.20(56.50,304.64) | Immune-mediated myocarditis | 5 | 63.30(25.25,158.69) |
| Secondary adrenocortical insufficiency | 24 | 120.41(76.68,189.07) | Thyroiditis | 9 | 53.37(27.02,105.40) |
| Immune-mediated hepatic disorder | 27 | 104.27(68.54,158.62) | Type 1 diabetes mellitus | 11 | 51.92(28.07,96.05) |
| Immune-mediated enterocolitis | 46 | 101.33(73.50,139.68) | Adrenal disorder | 4 | 47.81(17.31,132.09) |
| Immune-mediated encephalitis | 5 | 97.74(37.14,257.21) | Immune-mediated hepatitis | 6 | 43.05(18.83,98.45) |
| Immune-mediated nephritis | 5 | 97.74(37.14,257.21) | Colitis | 103 | 40.46(33.07,49.50) |
| Blood corticotrophin decreased | 4 | 85.62(29.38,249.54) | Myocarditis | 26 | 38.63(25.97,57.45) |
| Immune-mediated lung disease | 22 | 84.86(53.76,133.95) | Immune-mediated lung disease | 5 | 35.48(14.41,87.33) |
| Adrenocorticotropic hormone deficiency | 19 | 80.09(49.12,130.58) | Enterocolitis | 11 | 35.04(19.08,64.34) |
| Immune-mediated hepatitis | 15 | 79.52(45.89,137.79) | Autoimmune thyroiditis | 4 | 34.43(12.58,94.17) |
| Immune-mediated renal disorder | 3 | 79.31(23.23,270.72) | Lipase increased | 20 | 30.79(19.64,48.28) |
| Fulminant type 1 diabetes mellitus | 24 | 75.25(48.80,116.05) | Autoimmune hepatitis | 8 | 24.73(12.20,50.15) |
| Thyroiditis | 20 | 73.35(45.69,117.77) | Intestinal perforation | 15 | 23.80(14.20,39.88) |
| Endocrine disorder | 3 | 70.96(20.99,239.88) | Amylase increased | 7 | 23.19(10.90,49.32) |
| Silent thyroiditis | 3 | 70.96(20.99,239.88) | Encephalitis | 7 | 21.33(10.04,45.32) |
| Tumour pseudoprogression | 4 | 54.49(19.29,153.86) | Malignant neoplasm progression | 82 | 21.27(17.03,26.57) |
| Immune-mediated myocarditis | 10 | 54.23(28.12,104.58) | Hyperthyroidism | 17 | 20.20(12.45,32.77) |
| Adrenal disorder | 8 | 49.99(24.07,103.84) | Haemophagocytic lymphohistiocytosis | 7 | 19.57(9.22,41.55) |
| Adrenal insufficiency | 60 | 49.03(37.50,64.09) | Optic neuritis | 3 | 19.36(6.14,61.07) |
| Adrenocortical insufficiency acute | 6 | 42.16(18.25,97.40) | Peripheral motor neuropathy | 3 | 18.09(5.74,57.01) |

**Supplementary Table 9 Top 50 adverse events after exclusion of combinations for ipilimumab**

| PT | Case number | ROR(95% CI) | PRR(χ^2^) | IC(IC025) | EBGM(EBGM05) |
| --- | --- | --- | --- | --- | --- |
| Diarrhoea | 203 | 4.65(4.04,5.36) | 4.44(547.69) | 2.15(1.92) | 4.44(3.85) |
| Malignant neoplasm progression | 133 | 17.65(14.84,21.00) | 17.03(2001.09) | 4.08(3.67) | 16.95(14.25) |
| Colitis | 123 | 52.90(44.14,63.41) | 51.10(5959.22) | 5.65(4.91) | 50.38(42.03) |
| Death | 117 | 1.63(1.36,1.96) | 1.61(27.71) | 0.69(0.41) | 1.61(1.34) |
| Rash | 91 | 4.01(3.25,4.94) | 3.93(199.90) | 1.97(1.62) | 3.93(3.19) |
| Fatigue | 72 | 1.65(1.30,2.08) | 1.63(17.93) | 0.71(0.35) | 1.63(1.29) |
| Pruritus | 63 | 3.09(2.41,3.97) | 3.06(87.56) | 1.61(1.20) | 3.05(2.38) |
| Hypophysitis | 62 | 414.79(318.22,540.66) | 407.54(22538.00) | 8.51(5.37) | 365.39(280.33) |
| Off label use | 56 | 1.18(0.90,1.53) | 1.17(1.47) | 0.23(-0.16) | 1.17(0.90) |
| Nausea | 52 | 1.25(0.95,1.64) | 1.25(2.57) | 0.32(-0.09) | 1.25(0.95) |
| Asthenia | 46 | 1.49(1.11,1.99) | 1.48(7.22) | 0.57(0.13) | 1.48(1.11) |
| Decreased appetite | 46 | 2.27(1.70,3.03) | 2.25(32.16) | 1.17(0.71) | 2.25(1.68) |
| Pyrexia | 42 | 2.13(1.57,2.88) | 2.11(24.77) | 1.08(0.60) | 2.11(1.56) |
| Dehydration | 37 | 3.07(2.22,4.25) | 3.05(51.06) | 1.61(1.06) | 3.05(2.20) |
| Vomiting | 36 | 1.45(1.05,2.02) | 1.45(5.03) | 0.53(0.04) | 1.45(1.04) |
| Acute kidney injury | 31 | 1.56(1.10,2.22) | 1.56(6.20) | 0.64(0.10) | 1.56(1.09) |
| General physical health deterioration | 29 | 3.08(2.14,4.44) | 3.06(40.34) | 1.61(0.99) | 3.06(2.12) |
| Pneumonia | 29 | 0.97(0.67,1.40) | 0.97(0.02) | -0.04(-0.57) | 0.97(0.67) |
| Muscular weakness | 29 | 3.67(2.54,5.28) | 3.64(55.71) | 1.86(1.21) | 3.64(2.53) |
| Dyspnoea | 28 | 0.66(0.46,0.96) | 0.66(4.84) | -0.59(-1.11) | 0.66(0.46) |
| Weight decreased | 28 | 1.48(1.02,2.14) | 1.47(4.29) | 0.56(0.00) | 1.47(1.02) |
| Enterocolitis | 28 | 60.81(41.79,88.48) | 60.34(1606.62) | 5.89(3.76) | 59.34(40.78) |
| Intestinal perforation | 28 | 38.34(26.38,55.72) | 38.05(999.43) | 5.23(3.51) | 37.65(25.91) |
| Hyponatraemia | 26 | 3.52(2.40,5.18) | 3.51(46.62) | 1.81(1.12) | 3.50(2.38) |
| Sepsis | 26 | 2.88(1.96,4.24) | 2.87(31.71) | 1.52(0.87) | 2.87(1.95) |
| Hypotension | 25 | 1.47(0.99,2.17) | 1.46(3.69) | 0.55(-0.04) | 1.46(0.99) |
| Headache | 25 | 0.96(0.65,1.42) | 0.96(0.04) | -0.06(-0.62) | 0.96(0.65) |
| Malaise | 24 | 0.88(0.59,1.31) | 0.88(0.41) | -0.19(-0.76) | 0.88(0.59) |
| Intentional product use issue | 24 | 5.31(3.56,7.94) | 5.28(83.34) | 2.40(1.59) | 5.28(3.53) |
| Anaemia | 23 | 1.26(0.84,1.90) | 1.26(1.22) | 0.33(-0.28) | 1.26(0.83) |
| Abdominal pain | 23 | 1.95(1.30,2.94) | 1.95(10.61) | 0.96(0.31) | 1.95(1.29) |
| Adrenal insufficiency | 22 | 28.33(18.60,43.15) | 28.16(571.76) | 4.80(3.08) | 27.94(18.34) |
| Immune-mediated enterocolitis | 22 | 71.88(47.07,109.77) | 71.44(1497.75) | 6.13(3.52) | 70.04(45.86) |
| Hypothyroidism | 20 | 9.56(6.16,14.84) | 9.51(152.00) | 3.25(2.12) | 9.49(6.11) |
| Hypopituitarism | 19 | 122.59(77.50,193.91) | 121.93(2202.79) | 6.88(3.45) | 117.89(74.53) |
| Prescribed overdose | 19 | 22.82(14.52,35.88) | 22.71(391.85) | 4.50(2.79) | 22.57(14.36) |
| Rash pruritic | 19 | 5.52(3.52,8.67) | 5.50(69.86) | 2.46(1.52) | 5.49(3.50) |
| Neoplasm malignant | 18 | 4.95(3.11,7.86) | 4.92(56.28) | 2.30(1.36) | 4.92(3.09) |
| Pneumonitis | 17 | 7.43(4.61,11.98) | 7.40(94.01) | 2.89(1.76) | 7.39(4.59) |
| Arthralgia | 16 | 0.73(0.45,1.20) | 0.74(1.53) | -0.44(-1.12) | 0.74(0.45) |
| Adverse event | 16 | 4.58(2.80,7.48) | 4.56(44.47) | 2.19(1.21) | 4.56(2.79) |
| Back pain | 15 | 1.00(0.60,1.65) | 1.00(0.00) | -0.01(-0.73) | 1.00(0.60) |
| Autoimmune hepatitis | 15 | 37.69(22.64,62.75) | 37.53(527.86) | 5.22(2.78) | 37.15(22.31) |
| Hepatitis | 15 | 9.77(5.88,16.24) | 9.73(117.29) | 3.28(1.93) | 9.71(5.84) |
| Large intestine perforation | 15 | 25.01(15.03,41.60) | 24.91(341.83) | 4.63(2.59) | 24.74(14.87) |
| Pain | 13 | 0.50(0.29,0.86) | 0.50(6.56) | -1.00(-1.72) | 0.50(0.29) |
| Pleural effusion | 13 | 2.21(1.28,3.80) | 2.20(8.54) | 1.14(0.25) | 2.20(1.28) |
| Constipation | 12 | 0.77(0.44,1.35) | 0.77(0.84) | -0.38(-1.16) | 0.77(0.44) |
| Fall | 12 | 0.34(0.19,0.59) | 0.34(15.64) | -1.56(-2.29) | 0.34(0.19) |
| Chills | 12 | 1.70(0.96,3.00) | 1.70(3.45) | 0.76(-0.11) | 1.70(0.96) |

**Supplementary Table 10 Time-to-onset analysis for ipilimumab-related signals using the Weibull distribution test.**

| Events | N | TTO (days) | Weibull distribution | | Failure type |
| --- | --- | --- | --- | --- | --- |
|  |  |  | Scale parameter | Shape parameter |  |
|  |  | Median (IQR) | α(95% CI) | β(95% CI) |  |
| Overall | 3329 | 42.00(18.00,80.00) | 70.56(67.51,73.75) | 0.83(0.81,0.85) | Early failure |
| Endocrine disorders | 505 | 50.00(22.00,84.00) | 72.99(66.17 ,80.51 ) | 0.95(0.90,1.01) | Random failure |
| Metabolism and nutrition disorders | 632 | 43.00(21.00,79.00) | 70.74(64.50 ,77.57 ) | 0.90(0.85,0.96) | Early failure |
| Vascular disorders | 212 | 41.00(14.00,71.00) | 63.11(53.43 ,74.55 ) | 0.87(0.79,0.96) | Early failure |
| Gastrointestinal disorders | 1026 | 40.00(19.00,69.00) | 59.45(55.27 ,63.94 ) | 0.90(0.87,0.94) | Early failure |
| Injury, poisoning and procedural complications | 326 | 39.00(12.00,73.00) | 66.88(57.94 ,77.18 ) | 0.84(0.77,0.91) | Early failure |
| Infections and infestations | 667 | 38.00(14.00,79.00) | 65.31(58.73 ,72.63 ) | 0.76(0.72,0.81) | Early failure |
| Musculoskeletal and connective tissue disorders | 288 | 37.00(17.00,71.00) | 63.79(55.44 ,73.40 ) | 0.89(0.82,0.97) | Early failure |
| Blood and lymphatic system disorders | 274 | 36.00(11.00,63.00) | 52.71(45.40 ,61.19 ) | 0.85(0.78,0.93) | Early failure |
| Nervous system disorders | 445 | 35.00(16.00,70.00) | 61.02(54.27 ,68.62 ) | 0.86(0.80,0.92) | Early failure |
| General disorders and administration site conditions | 804 | 34.00(11.00,70.00) | 58.41(53.15 ,64.20 ) | 0.80(0.76,0.84) | Early failure |
| Respiratory, thoracic and mediastinal disorders | 615 | 33.00(12.00,72.00) | 57.59(51.72 ,64.13 ) | 0.79(0.74,0.83) | Early failure |
| Cardiac disorders | 290 | 32.00(15.00,72.00) | 60.71(51.92 ,70.99 ) | 0.79(0.73,0.85) | Early failure |
| Neoplasms benign, malignant and unspecified | 554 | 31.00(12.00,66.00) | 56.45(50.34 ,63.30 ) | 0.78(0.74,0.83) | Early failure |
| Renal and urinary disorders | 284 | 30.00(17.00,65.00) | 53.80(46.80 ,61.85 ) | 0.89(0.82,0.97) | Early failure |
| Hepatobiliary disorders | 414 | 29.00(13.00,65.00) | 50.25(44.84 ,56.31 ) | 0.91(0.85,0.98) | Early failure |
| Eye disorders | 81 | 28.00(13.00,52.00) | 54.36(40.47 ,73.01 ) | 0.80(0.69,0.94) | Early failure |
| Psychiatric disorders | 103 | 27.00(7.00,70.00) | 57.34(43.38 ,75.81 ) | 0.77(0.67,0.90) | Early failure |
| Investigations | 434 | 26.00(11.00,61.00) | 51.25(45.19 ,58.12 ) | 0.82(0.76,0.88) | Early failure |
| Immune system disorders | 122 | 21.50(7.00,57.00) | 53.14(39.97 ,70.65 ) | 0.70(0.61,0.79) | Early failure |
| Skin and subcutaneous tissue disorders | 431 | 19.00(6.00,43.00) | 36.97(31.81 ,42.95 ) | 0.69(0.64,0.73) | Early failure |

**Abbreviations**: N, number of cases with available time-to-onset; IQR, interquartile range; TTO, Time-to-onset.


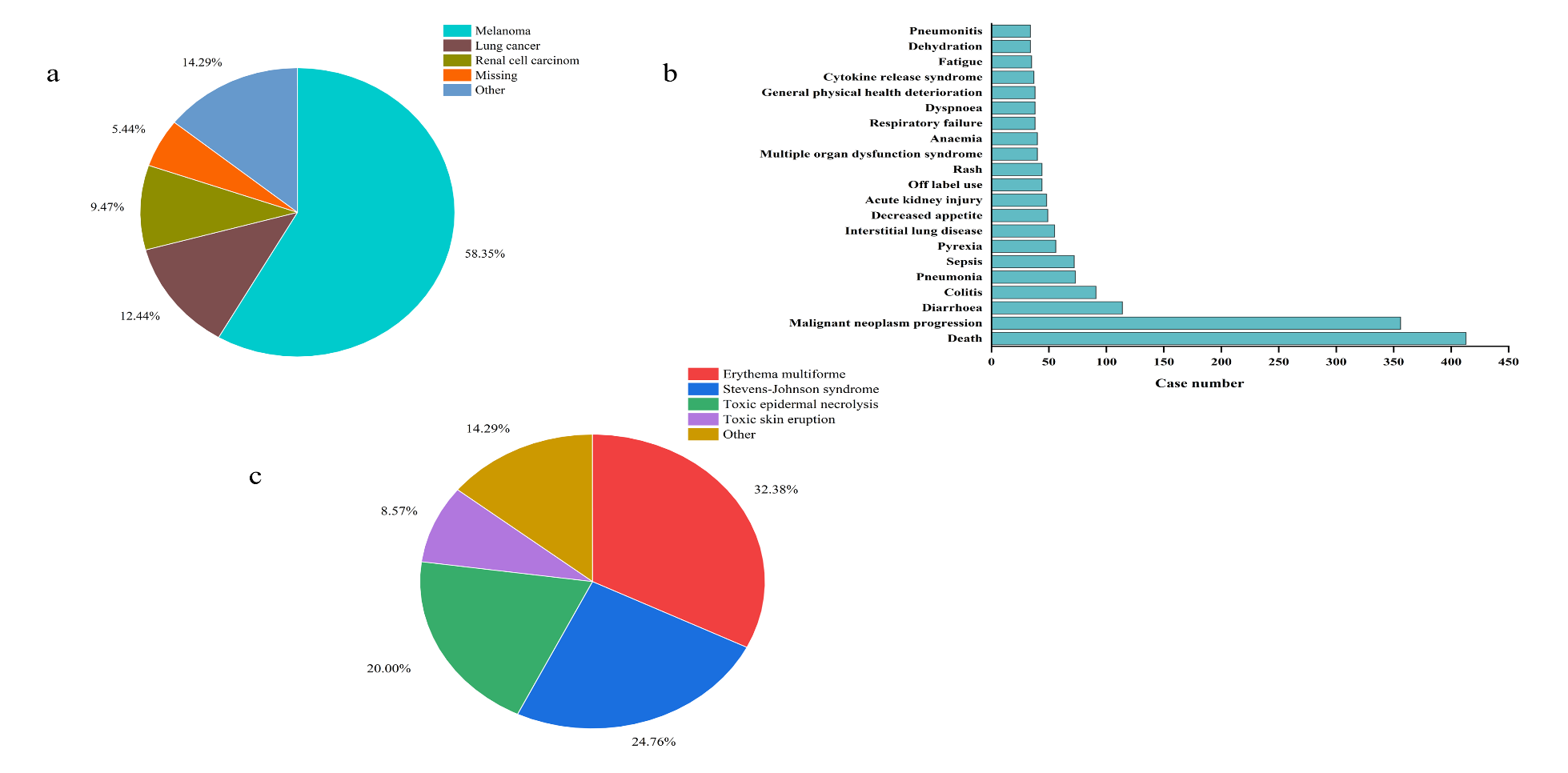


**Supplementary Figure 2** **Distribution of adverse events associated with ipilimumab (a)** **Indication distribution of gastrointestinal disorder adverse events associated with ipilimumab.(b)** **Adverse events experienced by patients who died in association with ipilimumab.(c)** **Distribution of serious skin adverse events associated with ipilimumab**
